# Self‐Propelling Hybrid Gels Incorporating an Active Self‐Assembled, Low‐Molecular‐Weight Gelator

**DOI:** 10.1002/chem.202102472

**Published:** 2021-09-09

**Authors:** Carmen C. Piras, David K. Smith

**Affiliations:** ^1^ Department of Chemistry University of York Heslington York YO10 5DD UK

**Keywords:** gels, mobility, self-assembly, self-propulsion, supramolecular chemistry

## Abstract

Hybrid gel beads based on combining a low‐molecular‐weight gelator (LMWG) with a polymer gelator (PG) demonstrate an enhanced ability to self‐propel in water, with the LMWG playing an active role. Hybrid gel beads were loaded with ethanol and shown to move in water owing to the Marangoni effect changes in surface tension caused by the expulsion of ethanol – smaller beads move farther and faster than larger beads. Flat shapes of the hybrid gel were cut using a “stamp” – circles moved the furthest, whereas stars showed more rotation on their own axes. Comparing hybrid LMWG/PG gel beads with PG‐only beads demonstrated that the LMWG speeds up the beads, enhancing the rate of self‐propulsion. Self‐assembly of the LMWG into a “solid‐like” network prevents its leaching from the gel. The LMWG also retains its own unique function – specifically, remediating methylene blue pollutant dye from basic water as a result of noncovalent interactions. The mobile hybrid beads accumulate this dye more effectively than PG‐only beads. Self‐propelling gel beads have potential applications in removal/delivery of active agents in environmental or biological settings. The ability of self‐assembling LMWGs to enhance mobility and control removal/delivery suggests that adding them to self‐propelling systems can add significant value.

## Graphical abstract

**Motion capture**: Gel beads that incorporate a self‐assembled low‐molecular‐weight gelator (LMWG) into an agarose polymer gel particle are reported; when loaded with ethanol and placed in water, they self‐propel as a result of the Marangoni effect. The presence of the LMWG significantly increases their mobility and endows them with unique functions, such as enhanced ability to adsorb a pollutant dye.

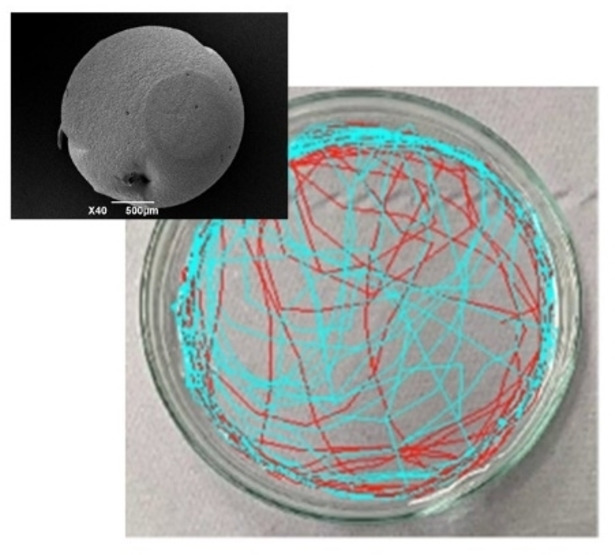

## Introduction

Self‐assembled hydrogels are fascinating, responsive soft materials with potential applications ranging from environmental regeneration to tissue engineering.^[1]^ They self‐assemble from low‐molecular‐weight gelators (LMWGs) and hence benefit from the synthetic programmability of these building blocks and the reversibility of the assembly step.^[2]^ However, self‐assembled gels are often weak materials, and this can make them difficult to physically manipulate. There has been increasing interest in achieving spatial and temporal control over such materials to access new forms of behaviour and types of application.^[3]^ One strategy is to form hybrid multicomponent systems with other LMWGs or indeed with polymer gelators (PGs), which can impart some robustness onto the system.^[4]^ For example, it has recently been demonstrated that combining LMWGs with the PG calcium alginate can give rise to well‐defined gel beads of sizes ranging from several millimetres to 800 nm.^[5]^ There has also been increasing interest in dynamic diffusion processes within self‐assembled gel matrices.^[6]^

One innovative way to spatially and temporally control LMWGs is to develop gels that can physically move in programmable ways. Such actuating systems can be considered as hydrogel machines and are an emergent area of intense interest.^[7]^ Most studies have focussed on polymer gels rather than LMWGs,^[8]^ indeed reports of shape‐changing LMWG systems are exceptionally rare.^[9]^ In addition to shape‐changing, there has also been interest in polymer systems capable of self‐propulsion.^[10]^ There are a number of strategies for driving self‐propelled systems. In one approach, a chemical fuel is used – for example, systems with embedded catalysts can move in aqueous hydrogen peroxide as a result of the chemical breakdown of H_2_O_2_ giving rise to an inhomogeneous concentration around the surface of the particle.^[11]^ Alternatively, particle motion can be induced by the imposition of an external field – often electrical or magnetic in nature.^[12]^ A different approach makes use of physical processes, such as the Marangoni effect, in which a gradient of surface tension is created, which leads to propulsion.^[13]^ Typically, surface tension gradients are created by inhomogeneously organised surfactant or co‐solvent.^[14]^ There is great interest in understanding how such processes can be controlled, and in coupling motion to other processes to yield non‐linear systems.^[15]^

Gels are ideal for creating self‐propelled machines as a result of their solvent compatibility, porous structures and ability to encapsulate and release molecules in a controlled manner. Motile gel beads with their own source of propulsion have potential uses in bio‐nanotechnology and medicine, where they can explore their surroundings, and deliver bioactive systems.^[16]^ Alternatively, they can be useful in environmental applications where they can physically explore confined spaces, removing, delivering or sensing chemical entities in their surroundings.^[17]^ Polymer gels have been widely explored in this regard, but surprisingly, to the best of our knowledge, self‐assembling LMWGs have not been incorporated into self‐propelling systems.

**Figure 1:**
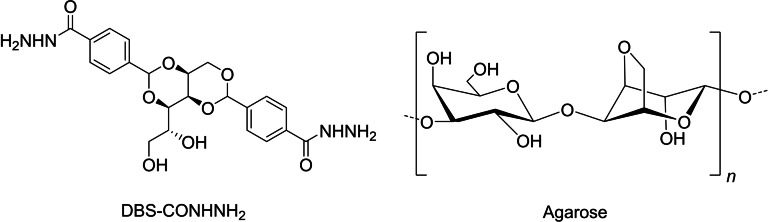
Chemical structures of low‐molecular‐weight gelator (LMWG) DBS‐CONHNH_2_ and polymer gelator (PG) agarose.

Having developed our innovative LMWG gel bead platform,^[5]^ we were interested in making this class of self‐assembled materials move. In this case, rather than using an alginate PG, we proposed to combine the LMWG DBS‐CONHNH_2_ with the PG agarose (Figure 1). This is a system we understand well in sample vials,^[18]^ but have not previously used to fabricate gel beads. Both gelators are thermally induced, and should simultaneously co‐assemble into gel beads with interwoven networks. Agarose is a good choice of PG in this case, as it is considered to be relatively inert, and has a high degree of robustness and mechanical strength.

## Results and Discussion

### DBS‐CONHNH_2_/agarose gel bead fabrication

**Figure 2:**
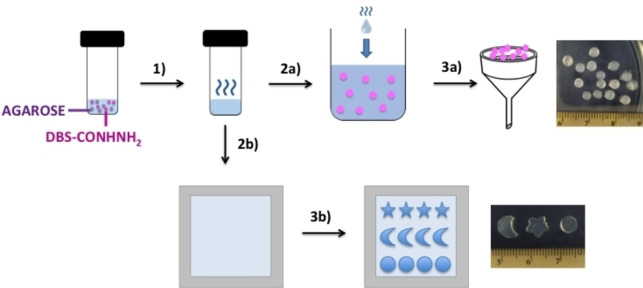
Schematic representation of the preparation of DBS‐CONHNH_2_/agarose gel beads and shaped gels. A DBS‐CONHNH_2_/agarose suspension is heated until complete dissolution of the two gelators (1). To obtain gel beads, the hot solution is added dropwise to a paraffin oil bath (2a), and the beads are isolated after 20 min by filtration (3a). To prepare shaped gels, the DBS‐CONHNH_2_/agarose hot solution is transferred into a glass tray (2b) and, once gelation is complete, shaped gels can be cut using small icing cutters (3b).

DBS‐CONHNH_2_ was synthesised in good yield by our previously reported method,^[19]^ whereas agarose is a commercially available polysaccharide. DBS‐CONHNH_2_/agarose two‐component gel beads were obtained by an emulsion method, using a 0.3 % *w*/*v* concentration of the LMWG and a 1.0 % *w*/*v* concentration of the PG. The two gelators were combined in water and the resulting suspension was heated until complete dissolution. The hot solution was then added dropwise to paraffin oil (20 μL drops) to give spherical gel beads on cooling (3.0–3.6 mm diameter; Figure 2, Figure S1 in the Supporting Information). To facilitate gelation, the paraffin oil was kept in an ice bath.

### DBS‐CONHNH_2_/agarose gel bead characterisation

**Figure 3:**
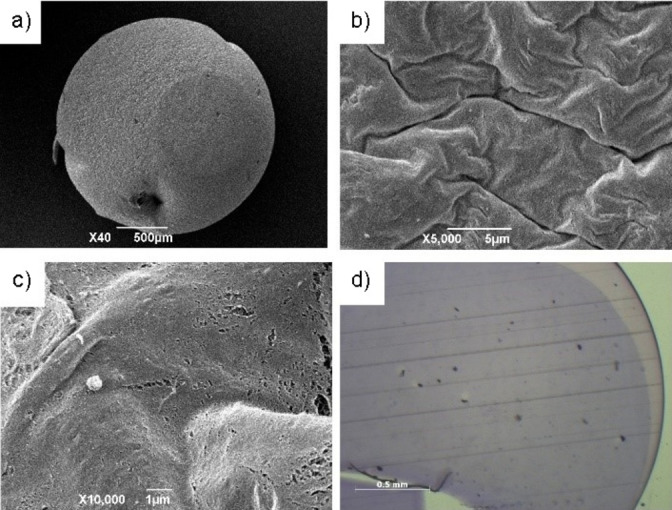
a) SEM image of a whole gel bead (scale bar: 500 μm); b) SEM image of the gel bead surface (scale bar: 5 μm). c) Cross‐section of a gel bead imaged by SEM (scale bar: 1 μm). d) Cross section of a gel bead embedded into resin, stained with toluidine blue and imaged by optical microscopy (scale bar: 500 μm).

Hybrid gels based on agarose and DBS‐CONHNH_2_ have been characterised in some detail previously.^[18]^ Having induced the simultaneous gelation of the two gelators with a thermal trigger, we reasoned that the two self‐assembled networks would be interwoven within the resulting gel beads. To confirm that the two gelators were incorporated into the gel beads in their self‐assembled form and obtain insight into the nanoscale morphology of the bead surface and cross‐section, we performed SEM. The DBS‐CONHNH_2_/agarose beads (Figure 3a) displayed a wrinkled surface (Figure 3b) and a densely packed fibrillar cross‐section (Figure 3c), confirming the expected self‐assembly. Optical microscopy of the gel beads cut in half, embedded into resin and stained with toluidine blue, showed a uniform distribution of the two networks within the gel beads (Figure 3d). The agarose‐only beads showed similar microscopy features (Figures S2 and S3).

To quantify the amount of LMWG loaded into each gel bead, we performed a simple ^1^H NMR experiment, as previously described for our DBS‐CONHNH_2_/alginate beads.^[5a]^ Ten gel beads prepared as described above were dried under high vacuum. ^1^H NMR of the solid beads dissolved in [D_6_]DMSO in the presence of a known amount of acetonitrile as an internal standard, allowed quantification of the LMWG by comparison of the integrals of the DBS‐CONHNH_2_ aromatic peaks to that of acetonitrile (Figure S4). This experiment indicated that each gel bead incorporated 99 % of the expected DBS‐CONHNH_2_. If, instead, the intact gel beads were studied by ^1^H NMR in D_2_O, no signal was observed for either agarose or DBS‐CONHNH_2_, demonstrating that both components are fully assembled into a solid‐like form. This is important because unlike blending a simple additive with the PG, the self‐assembly of the LMWG into a “solid‐like” nanoscale network limits leaching from the beads, and helps fix this network within the hybrid system.

The supramolecular interactions between the two gel networks within the beads were studied by IR spectroscopy (Figures S5 and S6). The O−H and N−H stretches of the LMWG appeared broadened in the presence of agarose, and the C=O band shifted from 1642 to 1665 cm^−1^. These shifts are consistent with the presence of supramolecular interactions between the LMWG and the PG networks and are similar to those previously observed for bulk samples of this hybrid gel.^[18]^

We also performed rheological characterisation of control agarose/DBS‐CONHNH_2_ gels made in sample vials in order to better understand the dual network nature of these hybrid hydrogel systems (Figures S7–S10, Table S3). In summary, the LMWG has a *G′* value of 800 Pa, the agarose PG has a *G′* value of 5960 Pa, and the combination of the two has a *G′* value of 29400 Pa. This clearly demonstrates that both gel networks are forming and interpenetrating to form a stiffer, more robust gel.

### DBS‐CONHNH_2_/agarose gel beads in motion

We then loaded the gel beads with EtOH to induce spontaneous motion in water by exploiting the Marangoni effect. In principle, as ethanol diffuses out of the beads, it changes the local surface tension close to the bead, leading to self‐propulsion. Such effects are understood in polymer gel systems, but in this study, we were interested in determining the potential benefits of incorporating an LMWG into PG beads.

**Figure 4:**
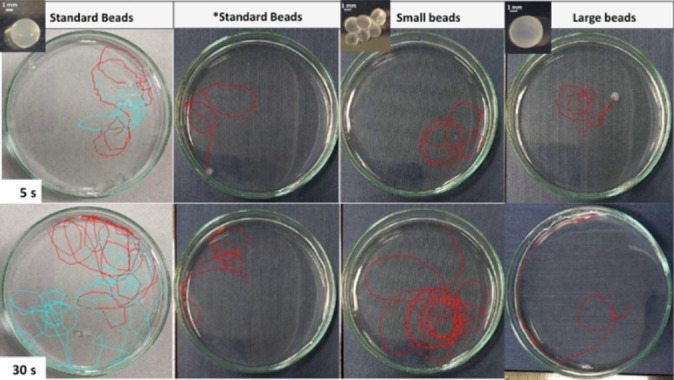
Trajectories travelled in 5 and 30 s (upper and lower images, respectively) by different types of DBS‐CONHNH_2_/agarose hydrogel beads: standard beads (3.0–3.5 mm diameter; two different trajectories are shown), small beads (1.5–2.0 mm diameter) and large beads (4.0–4.5 mm diameter). *Standard beads for which only half of the gel bead was immersed in EtOH for 15 min.

EtOH‐loaded beads were obtained by immersing them in EtOH for 24 h. When transferred into a small petri dish (8 cm diameter) filled with water, they displayed spontaneous motion for a maximum of 10–15 mins (“fast” for the first 1–2 mins then progressively slowing down. If the beads were not loaded with ethanol, no motion was observed (see the Supporting Videos). In general terms, the beads followed a random trajectory from the centre of the petri dish, where they were loaded, often ultimately ending up moving along the circumference (Figures 4 and S11).

First, we studied the effect of gel bead diameter on the total distance travelled after 5 and 30 s. Beads of different diameters were prepared by varying the drop volumes of the DBS‐CONHNH_2_/agarose hot solution added to the paraffin oil bath. Our standard gel beads (3.0–3.6 mm diameter) were prepared by using 20 μL droplet volumes. To obtain larger gel beads (4.0–4.5 mm diameter), we increased the droplet volume to 30 μL, whereas smaller beads (1.5–2.5 mm diameter) were prepared using 10 μL droplets. We refer to hybrid gel beads with diameters of 1.5–2.5 mm, 3.0–3.6 mm and 4.0–4.5 mm as “small”, “standard” and “large” respectively (Figure S1, Table S1).

**Figure 5:**
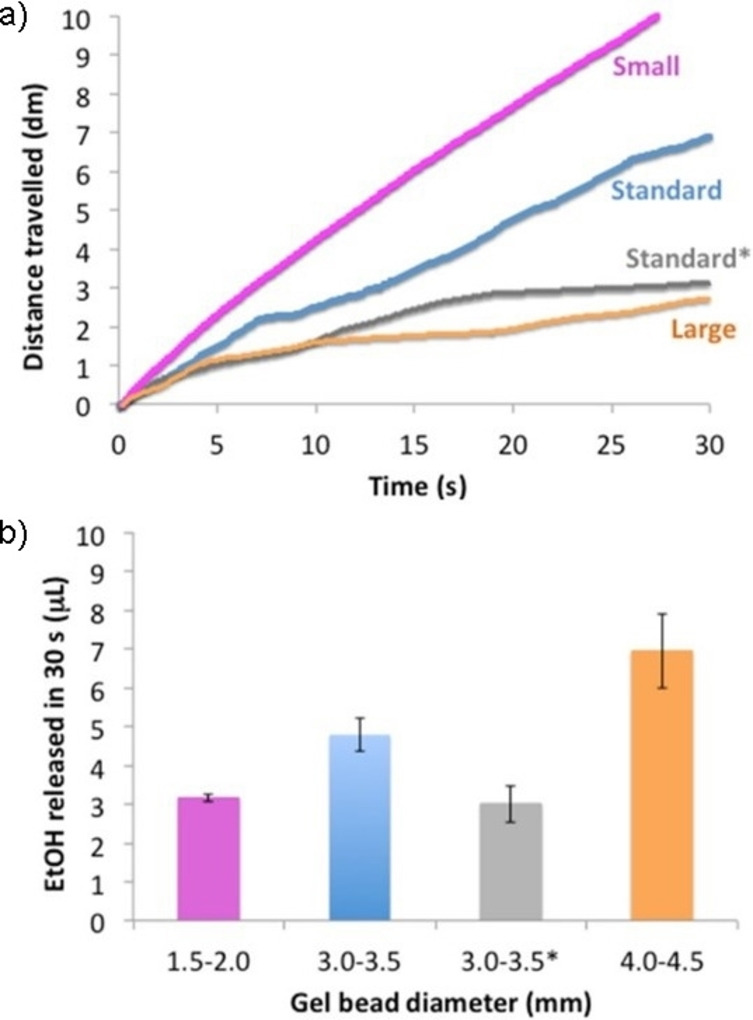
a) Distance travelled over time by DBS‐CONHNH_2_/agarose standard (blue and grey*), small (pink) and large gel beads (orange). b) EtOH released after 30 s by DBS‐CONHNH_2_/agarose gel beads of different diameters. *Only half gel bead was immersed in EtOH for 15 min.

The data indicate that the gel bead diameter significantly influences the total distance travelled and hence their average speed in the first 5 and 30 s (Figures S12–S14, Table S4). Initially, the standard and smaller gel beads showed similar behaviour, travelling 25–26 cm after 5 s at an average speed of about 5 cm/s (Table S4). The larger gel beads were significantly slower (average speed, 3.1 cm/s) and after 5 s travelled a shorter distance (15.7 cm; Table S4). After 30 s, the smaller gel beads had travelled a longer total distance (ca. 102 cm, Figure 5a, pink line) than the standard and larger gel beads (70.1 and 27.3 cm respectively, Figure 5a blue and orange lines). The average speed over 30 s was therefore higher for the smaller beads (3.4 cm/s) compared to the standard (2.3 cm/s) and the larger beads (0.9 cm/s). This indicates a significant impact of bead diameter on mobility.

To verify if the different motion was related to the EtOH released by each gel bead type, we quantified the amount of EtOH being released by the different beads using an enzymatic assay (EtOH assay, Sigma–Aldrich). This study was performed by immersing the different gel beads loaded with EtOH in water (5 mL) and subsequently analysing the EtOH content of the water after 30 s, 60 s and 24 h. After 30 seconds, the larger gel beads released a greater amount of EtOH (ca. 6.95 μL/bead, Figure 5b), followed by the standard beads (4.79 μL/bead) and the smaller beads (ca. 3.16 μL/bead). These differences were replicated at 60 s and 24 h (Table S4, Figures S15–S17). The greatest amount of EtOH was, as expected, released by the larger gel beads, however these displayed the slowest motion. The smaller beads are therefore significantly more mobile in spite of releasing less EtOH, and we therefore conclude that the enhanced mobility of the smaller beads is a function of their lower weight and smaller size.

To determine whether the amount of EtOH loaded into the beads could influence the motion in water of gel beads of the same size, we compared the motion in water of standard gel beads (3.0–3.5 mm diameter) that were immersed in EtOH for 24 h, with the behaviour of standard gel beads that were half‐immersed in EtOH for only 15 min (Figure S1). This lowered ethanol loading, the fully loaded gel beads released 8.62 μL/bead over 60 s, whereas the half‐loaded beads only released 6.15 μL/bead. In the first 30 s (Table S4), the half‐loaded beads travelled a significantly shorter distance of 31.4 cm (compared to 70.1 cm for the fully loaded beads) at a lower average speed of 1.0 cm/s (compared with 2.3 cm/s). This confirms that the EtOH loading does influence the self‐propulsion of beads, when beads with equivalent diameters and weights are compared (Section S10.2 in the Supporting Information).

We had wondered whether half‐immersing the beads in EtOH for loading may influence the directionality of motion by preferentially loading one half of the gel bead with ethanol. It is well‐known that “Janus” particles can exhibit a degree of controlled motion.^[20]^ However, we did not observe any apparent control over motion, with a similar “random walk” of the beads being observed for the half‐immersed beads as for fully loaded ones – presumably diffusion of EtOH within the bead prevents asymmetry from being induced using this simplistic approach. We suggest that in the future, the use of etching to shape the gel bead may be a way of inducing greater directionality of movement

Next, we explored whether the gel shape could affect motion – such effects are indeed known in self‐propelling systems.[^14a^, ^21^] We prepared differently shaped DBS‐CONHNH_2_/agarose gels (0.3 % *w*/*v* of DBS‐CONHNH_2_ and 1.0 % *w*/*v* of agarose) using small “icing cutters” (i. e., stars, crescents and circles) to a hybrid gel prepared in a 5×5 cm square tray (5 mL volume, see Figure 2). The shapes had broadly similar overall dimensions and weights (Figure S18, Table S6), as this is clearly a factor in controlling mobility (see above). This cutting process gives “2D” flat shapes, unlike the 3D “spheres” described above.

**Figure 6:**
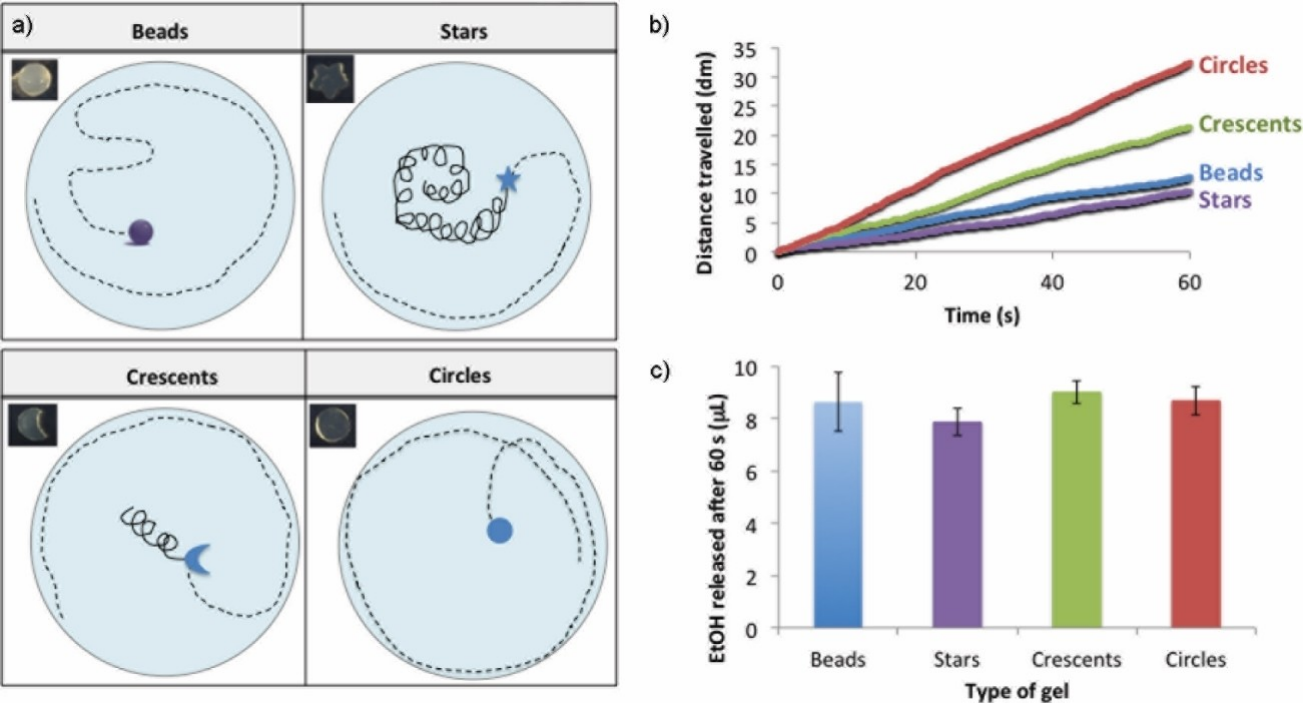
a) Schematic representation of the trajectories travelled by the different gel types. b) Distance travelled by DBS‐CONHNH_2_/agarose gel beads (3.0–3.5 mm diameter and shaped gels. c) EtOH released in 60 s from DBS‐CONHNH_2_/agarose gels of different shapes.

We loaded the shaped gels with EtOH and once again studied them in water, where they moved for a maximum of 20–30 min (“fast” for the first 1–2 mins, then progressively slowing down; see the Supporting Videos). The total distance travelled by the shaped gels after 5 s was similar for the different shapes and also for the gel beads (17–26 cm, Table S7, Figures S20 and S21). The circle and the crescent‐shaped gels were the fastest, with an average speed of 5.8 and 5.6 cm/s, followed by the gel beads (5.2 cm/s) and the stars (4.3 cm/s; Table S7, Figures S22 and S23). After 60 s, however, a remarkable difference had opened up in the total distance travelled by the different gels. The circular gels travelled the furthest (324 cm, Figure 6b) at an average speed of 5.4 cm/s, followed by the crescent‐shaped gels, which travelled 215 cm at 3.6 cm/s (Figure 6b). The gel beads and the stars travelled smaller distances (127 and 104 cm, respectively, Figure 6b) at lower average speeds (2.1 and 1.7 cm/s, respectively, Table S7). The circular gels therefore maintain a speed of 10 body lengths per second over the first minute, a significant rate for a soft matter self‐propelled object, whereas for the star shaped gels, this falls to just 3 body lengths per second.

**Figure 7:**
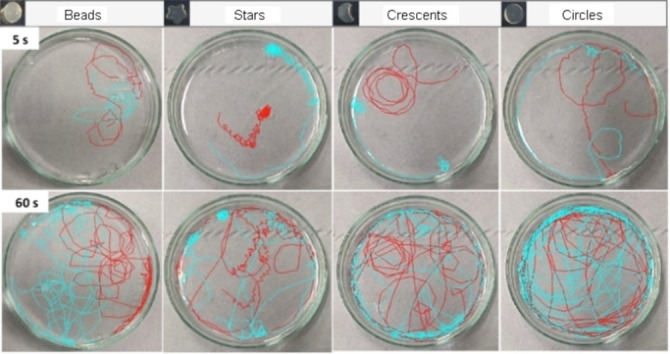
Trajectories travelled in 5 and 60 s by DBS‐CONHNH_2_/agarose hydrogel beads (3.0–3.5 mm diameter) and star‐shaped, crescent‐shaped and circular gels.

The differently shaped gels also showed different types of motions in water (Figures 6a and 7). The star‐shaped gels preferentially exhibited a rotational motion around their axis along with circular movements all over the petri dish surface and the circumference (Figures 6a and 7, Supporting Video 3). The crescent‐shaped gels preferentially moved in circles all over the petri dish surface and the circumference, and occasionally showed a rotational motion around their axis (Figures 6a and 7, Supporting Video 2). Conversely, the circular gels preferentially moved in circles all over the petri dish surface and the circumference without apparent rotational movement around their axis (Figures 6a and 7). We reasoned that the differences in speed and total distance travelled were primarily due to their shapes and related preferential motion types. For example, the star, which exhibits the largest amount of motion around its own rotational axis, moved the smallest distance of any of the shapes, whereas the crescent shape and especially the circle, which moved with less rotational motion, travelled much farther distances across the petri dish.

To determine whether the EtOH released by the differently shaped gels had an impact on their mobility, we assayed the shapes to quantify the amount of EtOH released in water (Figures 6c and S30–S32, Table S9). There were some differences in amounts of ethanol released, with slightly less ethanol being released from the stars than the crescents or circles. This might, in addition to the greater rotational motion, influence the lower mobility of the stars. However, conversely, the crescents released more ethanol than the circles, even though they do not move as far. In any case, the small differences in amounts of ethanol released are not reflected in the large differences in the mobility of the objects, therefore confirming that shape plays the primary role in controlling mobility.

Compared with the 2D cut shapes, the gel beads, which have 3D curvature, were intermediate in terms of mobility – similar to the stars, and significantly less than the crescents or circles. We suggest this may be because they are not so effective at releasing EtOH at the surface of the solvent bath compared to the flatter 2D shapes. It is this surface‐located ethanol which drives the Marangoni effect responsible for self‐propulsion.

Finally, and importantly, we compared the mobility of the different gel shapes based on the hybrid DBS‐CONHNH_2_/agarose gel, with the mobility of pure agarose gel shapes (Figures S21, S23, snd S25). This allowed us to determine the impact of the LMWG on self‐propulsion (Table 1). Although over the first 5 s, the mobility of both compositions was similar, over 60 s, the hybrid LMWG/PG gels move significantly faster and farther than the agarose‐only systems. Indeed, this difference in mobility is as much as a factor of two for the gel beads, where the hybrid DBS‐CONHNH_2_/agarose beads move at an average speed of 2.1 cm/s but the agarose‐only beads move at just 1.0 cm/s (Table 1, Figure S23). On average, for the different particles investigated here, the mobility is increased by 68 % by the presence of the LMWG. In this way, the presence of the self‐assembled LMWG appears to enhance the performance of these self‐propelling shaped gels.

**Table 1:** EtOH release after 60 s and 24 h from DBS‐CONHNH_2_/agarose and agarose gel beads and shaped gels.

Gels	Distance travelled in 60 s [cm]		Average speed [cm/s]		EtOH release after 60 s [μL]		EtOH release after 24 h [μL]	
	Hybrid	Agarose	Hybrid	Agarose	Hybrid	Agarose	Hybrid	Agarose
beads	127	61.0	2.1	1.0	8.63	6.35	19.97	19.39
stars	104	60.9	1.7	1.0	7.86	6.29	32.18	34.08
crescents	215	165	3.6	2.7	8.99	7.36	32.14	37.20
circles	324	206	5.4	3.4	8.67	6.65	31.33	28.08

To understand this difference in mobility, we determined the amount of EtOH released from the different objects (Table 1). In each case, after 60 s, the amount of ethanol released from the hybrid gel objects was significantly more than from agarose‐only objects (on average, 28 % more). Interestingly, however, after 24 h the amount of ethanol released was very similar for the hybrid gel objects and the agarose‐only objects (Table S9, Figures S30–S32). Indeed, averaged across all objects, 4 % less ethanol is released from the hybrid gels over 24 h. This indicates that although the total EtOH loading of the different objects is similar, irrespective of the presence of the LMWG, the presence of the self‐assembled network increases the initial rate of release of EtOH, and hence enhances the mobility. We suggest this likely reflects interactions between EtOH/H_2_O and the self‐assembled gel network that facilitate rapid EtOH release. Importantly, it should also be noted the self‐assembly of DBS‐CONHNH_2_ into a “solid‐like” network prevents its leaching from the gel beads – offering a significant advantage compared with just blending a simple small molecule into agarose gel beads in order to try and modify solvent release. In this way, the presence of this immobilised self‐assembled gel network acts to “turbo‐charge” the movement by enhancing the initial rate of ethanol release.

### Dye recovery

We wanted to demonstrate that the presence of the LMWG added function to these self‐propelled gel beads. We therefore focused on dye recovery/adsorption. Hydrogels are particularly suitable materials for this purpose, as they are porous, compatible with environmentally relevant aqueous media, and the nanostructured fibrillar materials have high effective surface areas capable of interactions with pollutant species.^[22]^ Most commonly, gels are used to passively adsorb waste such as dyes from water, or are applied in filtration mode. A gel that is capable of motion can potentially access regions that other gels could not reach.[^17a^, ^17b^, ^17c^] Mobile gels can therefore be considered as scavenger species, capable of using their mobility to drive chemical change with spatial resolution. We have previously demonstrated that DBS‐CONHNH_2_ can absorb dyes from water with the pH controlling the degree of dye uptake.[^19^, ^23^] In this study, we decided to use methylene blue as a model dye because it had the potential to be taken up effectively and visually by the gel – it is also a very widely used high‐volume industrially relevant cationic dye with some toxicity concerns.

**Figure 8:**
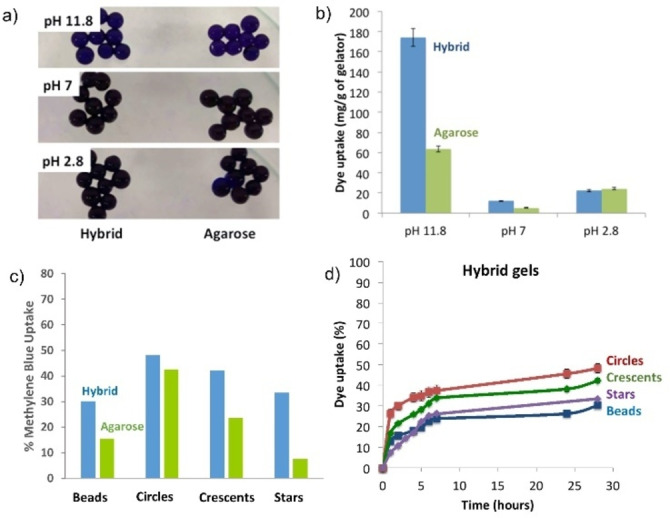
a) Photograph of DBS‐CONHNH_2_/agarose gel beads (prepared with 0.3 % *w*/*v* of LMWG and 1.0 % *w*/*v* of PG) and agarose gel beads (1.3 % *w*/*v*) after 24 h of exposure to a methylene blue solution, indicating blue colouration at pH 11.8 where methylene blue uptake is most significant. b) Methylene blue maximum uptake by DBS‐CONHNH_2_/agarose and agarose gel beads under different pH conditions. c) Methylene blue uptake [%] after 24 h at pH 11.8 by DBS‐CONHNH_2_/agarose and agarose gel beads and shaped gels loaded with EtOH and in motion. d) Methylene blue uptake [%] over time at pH 11.8 by DBS‐CONHNH_2_/agarose gel beads and shaped gels loaded with EtOH and in motion.

Initially, we tested the dye uptake achieved by static hybrid gel beads. Optimal methylene blue uptake of about 175 mg/g was achieved by the hybrid gel beads at basic pH, as previously demonstrated for the LMWG alone (Figures 8a,b and S34, Table S10).^[19]^ In addition, it was demonstrated that adding additional hybrid beads to the system, or removing hybrid gel beads and replacing them with fresh beads gave additional methylene blue remediation. In contrast, the agarose‐only gel beads only adsorbed 64 mg/g of methylene blue under the same conditions. DBS‐CONHNH_2_ is only poorly able to take up the dye at lower pH values,^[19]^ and this performance was also transferred across to these hybrid gel beads, which had methylene blue uptakes <25 mg/g under neutral and acidic conditions. At these lower pH values, the hybrid gel beads behaved the same as the agarose‐only gel beads (Figure 8b). These results demonstrate that the LMWG, DBS‐CONHNH_2_, retains its functional ability to remediate methylene blue under basic pH conditions within these hybrid gels and therefore adds its unique functionality to these shaped gel beads

We then studied the rate of dye uptake at different pH values (Table S11, Figure S33). Under basic conditions (pH 11.8), the hybrid gel beads took up approximately 70 mg/g of their maximum loading in the first hour, the next 70 mg/g over the following 4 h, and the remaining 35 mg/g over a 24 h period. To demonstrate the gel beads could be re‐used, after 24 h exposure to methylene blue, we washed them with basic NaOH solution until the colour was fully removed. On re‐using the gel beads, the performance in terms of total methylene blue uptake was actually even better than on the first run (Table S12, Figure S35), with the total uptake rising to approximately 280 mg/g by the fourth use. We suggest this is likely to be a result of the base‐washing process pre‐activating the DBS‐CONHNH_2_ network towards dye uptake. In each run the kinetics of dye uptake were similar. The agarose‐only beads slightly improved their performance on base washing, but even after four cycles of use, the maximum uptake was still only 125 mg/g – less effective than the DBS‐CONHNH_2_/agarose hybrid gel beads on their first use.

Finally, and importantly, we tested whether the moving beads could be used for dye recovery by performing a dye uptake experiment using the ethanol‐loaded mobile beads. The mobility of the gel particles in basic solution (pH 11.8–12.0) was similar to that in pure water (Figures S26–S29, Table S8). However, the movement was somewhat less consistent, with gel objects showing different rates of mobility over a 60 s timescale making it more difficult to draw conclusions about the effects of shapes. Pleasingly, the mobile ethanol‐loaded gel beads were able to remediate methylene blue from solution and importantly, in each case, the mobile beads with an embedded LMWG network very significantly outperformed the agarose‐only beads (Figures 8c and S37, Table S15). This clearly demonstrates that the unique functionality of the LMWG network is translated into the mobile beads and enhances their performance in this regard.

We compared the dye uptake by the differently shaped gels loaded with EtOH and moving within a petri dish of methylene blue (Figures 8d and S37, Table S15). In general, we found that dye uptake roughly correlates with mobility – dye uptake is most effective for the circles (48 % uptake) and crescents (42 % uptake), and less effective for the beads (30 % uptake) and stars (33 % uptake). This suggests that similar factors control dye uptake and ethanol release.

Interrogating the system in more detail, the moving gels (loaded with EtOH) were compared with static gels (without EtOH; Tables S14 and 15). Although the mobile gels and static gels eventually bind similar amounts of methylene blue, the mobile gels show slightly slower initial uptake rates than static gels, especially over the first 15 min while the beads are in most motion. We reasoned that this may be because initially, the moving gels release the loaded EtOH, an efflux process that may somewhat limit initial dye absorption. After that phase, they then start to absorb the dye more strongly. As such, the moving gel beads have potential to access difficult to reach locations through their motion where they could then absorb pollutant dyes. This approach could, in the future, be combined with magnetic bead technologies to facilitate bead recovery.

## Conclusions

In summary, we have reported a new family of supramolecular gel beads based on interwoven networks of DBS‐CONHNH_2_ and agarose. The beads were prepared by emulsion methods and have been fully characterised. Once the beads are loaded with ethanol, they are able to self‐propel in water as a result of the Marangoni effect. Smaller beads move farther and faster than larger beads. Furthermore, cutting the gels into shapes has a direct impact on the mobility, with motion being switched between translational modes and internal rotation. The presence of the self‐assembled, low‐molecular‐weight gelator network amplifies the Marangoni effect and significantly enhances the mobility of the shaped gels. This is a result of more rapid efflux of ethanol from the hybrid gel beads than the agarose‐only systems, which means that, even though they have similar total ethanol loadings, the system incorporating the LMWG is better able to harness it for rapid propulsion. This is a clear benefit of the hybrid gel approach.

The self‐assembled LMWG network retains its functionality in these gels, enhancing the remediation of the pollutant dye methylene blue under basic conditions, with the hybrid gel beads significantly outperforming agarose‐only objects. Dye uptake is reversible, and the gel beads can be reused multiple times. Importantly, the mobile ethanol‐loaded beads can remediate methylene blue from solution, and there is a general correlation between the ability of the beads to move and the amount of methylene blue removed, thus suggesting the LMWG controls both processes.

In terms of future perspective, we note that, in addition to binding dyes, this LMWG can bind precious heavy metals, remediating valuable waste streams and generating catalytic gel beads.[^5a^, ^24^] Self‐propelled catalytically active beads might be able to perform spatially resolved chemical reactions. Work to explore this is currently in progress. Given the ability to scale these beads down to the microscale, as we recently reported^[5c]^ and the ability of this LMWG to control the release of bio‐active agents,^[25]^ these mobile systems might also have applications in spatially resolved nanomedicine.

Thinking more broadly, this approach should not be limited to DBS‐CONHNH_2_. Different LMWGs have a wide range of different functions, and the ability to assemble these functional LMWGs into agarose gel beads and use the Marangoni Effect for self‐propulsion opens up a wide range of possibilities for the combination of LMWGs with mobile gel technology.

## Conflict of interest

The authors declare no conflict of interest.

## Supporting information

As a service to our authors and readers, this journal provides supporting information supplied by the authors. Such materials are peer reviewed and may be re‐organized for online delivery, but are not copy‐edited or typeset. Technical support issues arising from supporting information (other than missing files) should be addressed to the authors.

Supporting Information

Supporting Information

Supporting Information

Supporting Information

Supporting Information
