# Supplementary material for: Self‐Propelling Hybrid Gels Incorporating an Active Self‐Assembled, Low‐Molecular‐Weight Gelator
Source: Chemistry. 2021 Sep 9;27(58):14527–34. doi: 10.1002/chem.202102472 (PMC8597049; doi:10.1002/chem.202102472)
Supplement: Supplementary file 4 — Supporting Information [file CHEM-27-14527-s002.pdf]

# Chemistry–A European Journal

Supporting Information


## Self-Propelling Hybrid Gels Incorporating an Active Self-Assembled, Low-Molecular-Weight Gelator


Carmen C. Piras and David K. Smith*

# SUPPORTING INFORMATION


S1 General experimental methods

S2 Gel beads preparation

S3 Optical microscopy

S4 Scanning Electron Microscopy (SEM)

S5 NMR assay

S6 Infrared (IR) spectroscopy

S7 Thermal stability studies

S8 Rheology

S9 Gel beads motion studies

S10 Gel beads ethanol release studies

S11 Preparation of shaped gels

S12 Shaped gels motion studies

S13 Shaped gels ethanol release studies

S14 Dye uptake studies

S15 References

## S1. General Experimental Methods

All compounds used in synthesis and analysis were purchased from standard commercial suppliers and used as received. The synthesis of DBS-$CONHNH_2$ was performed in good yields applying previously reported methods.[1, 2] $^1$H NMR spectra were recorded using a Jeol 400 spectrometer ($^1$H 400 MHz). Samples were prepared in DMSO-$d_6$ and chemical shifts ($\delta$) are reported in parts per million (ppm). IR spectra of xerogels were recorded on a PerkinElmer Spectrum Two FT-IR spectrometer. Optical microscopy images were obtained using a Zeiss Axiocam camera on a Zeiss stereo microscope. SEM images were taken using a JEOL JSM-7600F field emission SEM. $T_{gel}$ values were obtained using a high precision thermoregulated oil bath using the tube inversion method and were recorded in triplicate. Rheology was measured on a Malvern Instruments Kinexus Pro+ Rheometer fitted with a 20 mm parallel plate geometry. The motion of gel beads and shaped gels was analysed using the Tracker Software. UV-vis spectra were collected on a BMG LabTech ClarioStar microplate reader (ethanol release studies) or on a UV-2401 PC spectrophotometer (dye uptake studies).

## S2. Gel beads preparation

*S2.1 DBS-$CONHNH_2$/agarose two-component gel beads.* DBS-$CONHNH_2$ (0.3% wt/vol) and agarose (1.0% wt/vol) were suspended in water (1 mL) and sonicated to help the dispersion of the solid particles. The resulting suspension was heated until complete dissolution of the two gelators. The hot solution was then added dropwise (20 μL/drop for standard gel beads with a 3.0-3.5 mm diameter) to ice cold paraffin oil (40 mL). Bigger or smaller gel beads (4.0-4.5 or 1.5-2.5 mm diameter respectively) were obtained by changing the droplets volume. The droplets were left undisturbed for 20 mins to allow the formation of the two gel networks. After this time, the gel beads were isolated and, to remove residual paraffin oil, they were immersed in petroleum ether (30 mL, 30 mins), then EtOH (30 mL, 30 mins) and finally water (30 mL, 30 mins). When necessary, the washings were performed multiple times. The gel beads were then stored in water.

*S2.2 Agarose gel beads.* Agarose (1.3% wt/vol) was suspended in water (1 mL) and then heated until complete dissolution. The hot solution was added dropwise (20 μL/drop for standard gel beads with a 3.0-3.5 mm diameter) to ice cold paraffin oil (40 mL). Bigger or smaller gel beads (4.0-4.5 or 1.5-

2.5 mm diameter respectively) were obtained by changing the droplets volume. The droplets were left undisturbed for 20 mins to allow the formation of the gel network. After this time, the gel beads were isolated and, to remove residual paraffin oil, they were immersed in petroleum ether (30 mL, 30 mins), then EtOH (30 mL, 30 mins) and finally water (30 mL, 30 mins). When necessary, washing was performed multiple times. The gel beads were then stored in water.

*S2.3 Preparation of gel beads for motion studies.* DBS-CONHNH$_2$/agarose and agarose beads for gel motion studies were prepared as described in the sections above and immersed in EtOH for 24 hours. DBS-CONHNH$_2$/agarose gel beads that were half immersed in EtOH for 15 mins, were kept in the solvent using a needle which was fixed on a peg on top of a small beacker containing 77 mL of EtOH.

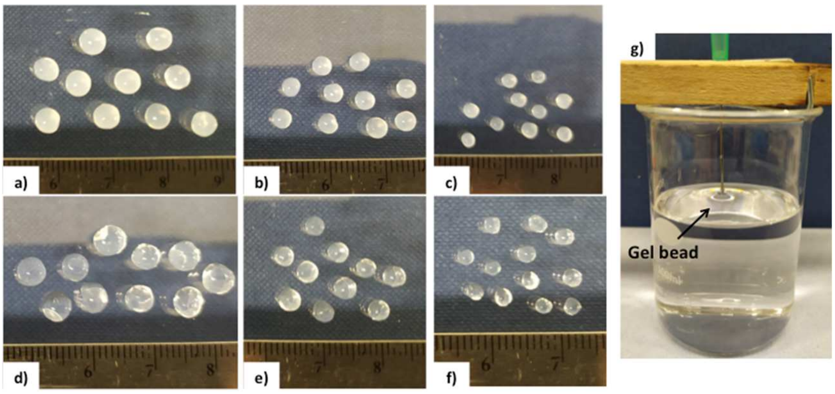


Figure S1. a-f) DBS-CONHNH$_2$/agarose gel beads (prepared with 0.3% wt/vol DBS-CONHNH$_2$ and 1.0% wt/vol agarose) and agarose gel beads (1.3% wt/vol) of different sizes: large (a and d respectively), standard (b and e respectively) and small (c and f respectively). g) Preparation of DBS-CONHNH$_2$/agarose gel beads half immersed in EtOH for 15 mins for gel motion studies.

Table S1. Diameter and weight of different DBS-CONHNH$_2$/agarose (prepared with 0.3% wt/vol DBS-CONHNH$_2$ and 1.0% wt/vol agarose) and agarose (1.3% wt/vol) gel beads.

| Gel | Average Diameter (mm) | Average Weight (mg) |
|---|---|---|
| Hybrid beads small | 2.59 +/- 0.14 | 8.28 +/- 0.41 |
| Hybrid beads standard | 3.51 +/- 0.16 | 22.32 +/- 0.44 |
| Hybrid beads large | 4.67 +/- 0.09 | 45.86 +/- 2.62 |
| Agarose beads small | 2.54 +/- 0.17 | 8.21 +/- 0.26 |
| Agarose beads standard | 3.61 +/- 0.22 | 22.38 +/- 0.25 |
| Agarose beads large | 4.97 +/- 0.35 | 46.22 +/- 2.63 |

*S2.4 DBS-CONHNH$_2$/agarose two-component gels in sample vials (for IR, thermal stability and rheology studies).* DBS-CONHNH$_2$ (0.3% wt/vol) and agarose (1.0% wt/vol) were suspended in water (1 mL) and sonicated to help the dispersion of the solid particles. The resulting suspension was heated until complete dissolution of the two gelators. The hot solution was then left undisturbed for a few hours to allow the formation of the gel networks.

*S2.5 Agarose gels in sample vials (for IR, thermal stability and rheology studies).* Agarose (1.3% wt/vol) was suspended in water (1 mL) and then heated until complete dissolution. The hot solution was left undisturbed for a few hours to allow the formation of the gel.

*S2.6 DBS-CONHNH$_2$ gels in sample vials (for IR, thermal stability, rheology and dye uptake studies).* DBS-CONHNH$_2$ (0.3% wt/vol) was suspended in water (1 mL). The suspension was sonicated to help the dispersion of the solid particles and then heated until complete dissolution of the compound. The sample was left undisturbed to cool, allowing gel formation in a few minutes.

## S3 Optical microscopy

Optical microscopy images were collected on a Zeiss stereo microscope. The gel beads were dehydrated through an ethanol series, then embedded in LR white resin. Sections were 1 μm thick. Once the section was dried on the slide, it was stained with Toluidine Blue (0.6% with 0.3% $Na_2CO_3$).

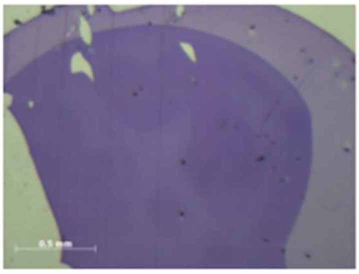

Figure S2. Optical microscopy image of agarose gel bead embedded in resin and stained with toluidine blue (scale bar 500 μm).

## S4 Scanning Electron Microscopy (SEM)

*S4.1 Preparation of samples for SEM.* Gel beads samples for SEM were obtained by freeze drying the gels on copper shim pieces. The freeze-dried samples were then mounted on stubs and the images recorded.

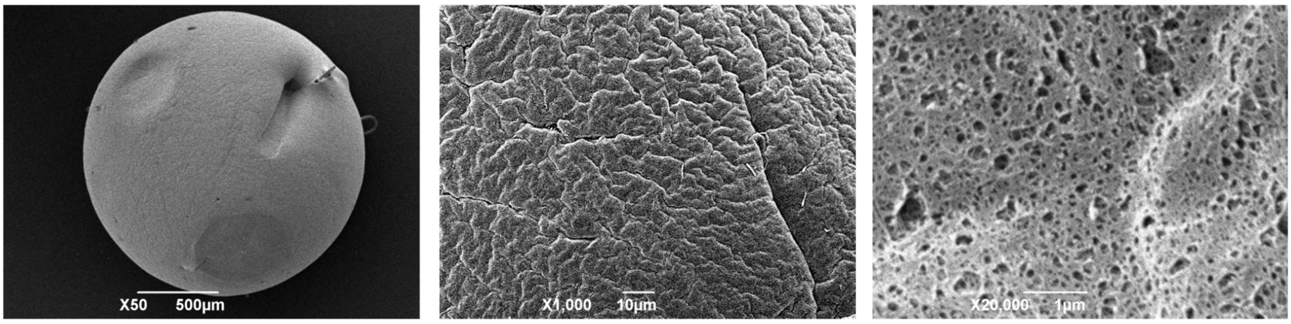


Figure S3. SEM images of agarose whole gel bead (left), surface (middle) and cross-section (right). Scale bars from left to right: 500, 10 and 1 µm.

## S5 NMR assay

*S5.1 Quantification of DBS-CONHNH$_2$ incorporated into DBS-CONHNH$_2$/agarose gel beads.* $^1$H NMR was employed to calculate the exact amount of DBS-CONHNH$_2$ incorporated into the DBS-CONHNH$_2$/agarose two-component gel beads prepared by emulsion. The gel beads used for this experiment were prepared by combining DBS-CONHNH$_2$ (0.3 % wt/vol) and agarose (1.0 % wt/vol) as described in Section S2.1. Ten gel beads were isolated and dried under high vacuum. The resulting solid was dissolved in DMSO-d$_6$ (0.7 mL), and acetonitrile (3.0 µL) was added as an internal standard. To make sure that all the DBS-CONHNH$_2$ was dissolved, the sample was ground and then sonicated for 30 min. The $^1$H NMR spectrum was recorded and the concentration of the LMWG calculated by comparison of the integrals of relevant peaks (DBS-CONHNH$_2$ aromatic peaks $\delta$ = 7.53 and 7.83 ppm) to that of acetonitrile ($\delta$ = 2.09 ppm). To ensure the results were reproducible, this experiment was performed on two different batches of gel beads.

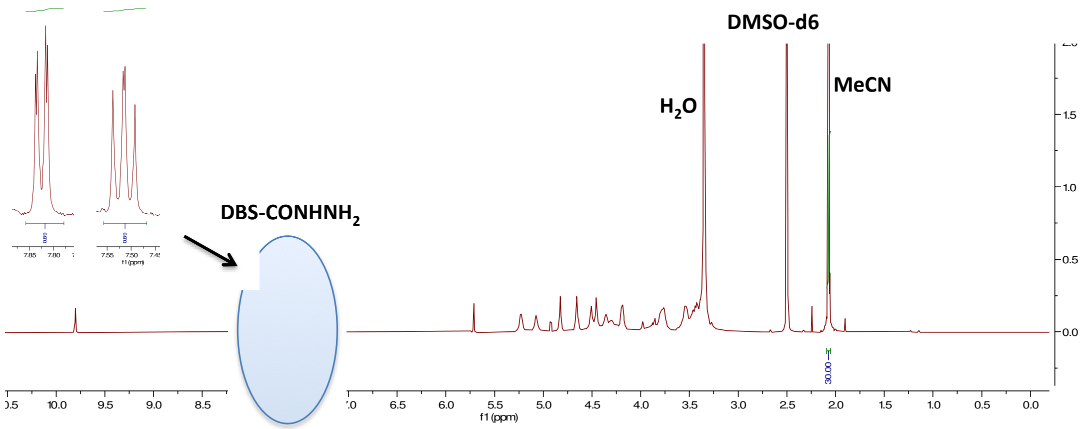


Figure S4. $^1$H NMR of ten dried DBS-CONHNH$_2$/agarose gel beads prepared by emulsion.

## S6 Infrared (IR) spectroscopy

Xerogel samples for IR were prepared by removing the solvent from gel beads and gels prepared in sample vials under high vacuum. A small amount of the resulting powder was placed into the infrared spectrophotometer and the spectra recorded in the range of 450-4000 cm$^{-1}$.

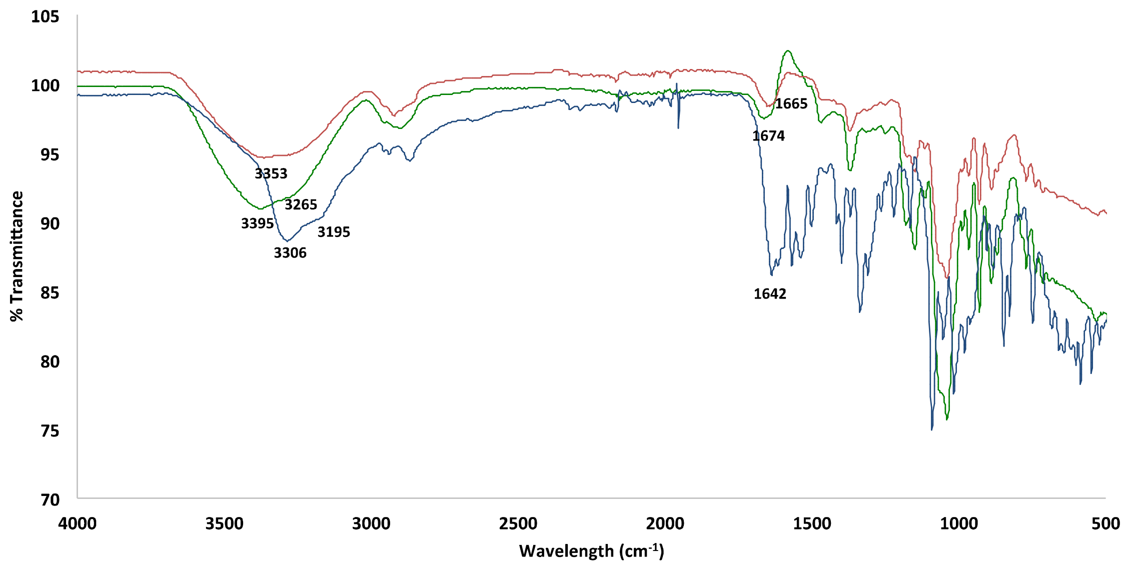


Figure S5. IR spectra of xerogels obtained from DBS-CONHNH$_2$ gel (0.3% wt/vol, blue line), agarose gel beads (1.3% wt/vol, green line) and DBS-CONHNH$_2$/agarose two-component gel beads containing 0.3% wt/vol of DBS-CONHNH$_2$ and 1.0% wt/vol agarose (red line).

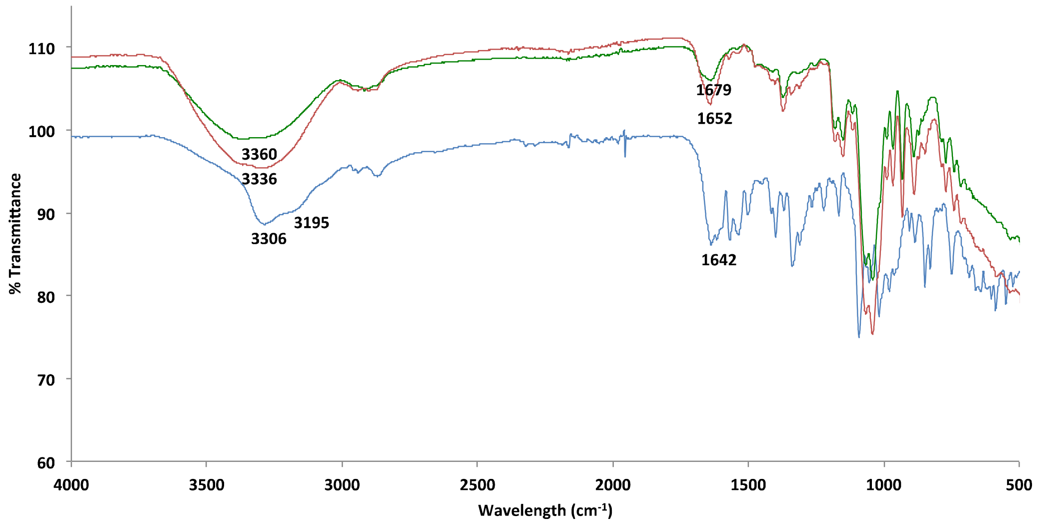


Figure S6. IR spectra of xerogels obtained from DBS-CONHNH$_2$ (0.3% wt/vol, blue line), agarose (1.3 % wt/vol, green line) and DBS-CONHNH$_2$/agarose (0.3% wt/vol of DBS-CONHNH$_2$ and 1.0% wt/vol agarose, red line) gels prepared in sample vials.

## S7. Thermal stability studies ($T_{gel}$ determination)

*S7.1 $T_{gel}$ determination method.* The gels for $T_{gel}$ determination were prepared as described in Section S2. All the gels were placed in a high precision thermoregulated oil bath with an initial temperature of 25 °C. The temperature was set to increase of 1 °C/ min until 100 °C were reached. Every minute the gels were checked by tube inversion method and $T_{gel}$ was considered as the temperature at which the gel began to run down the sides of the vial. These experiments were performed in triplicate to ensure reproducibility and average is reported.

*S7.2 $T_{gel}$ values*

Table S2. $T_{gel}$ values of gels formed by individual gelators and the DBS-CONHNH$_2$/agarose gel.

| GEL (1 mL total volume) | CONCENTRATION OF DBS-CONHNH$_2$ | CONCENTRATION OF AGAROSE | $T_{gel}$ |
|---|---|---|---|
| DBS-CONHNH$_2$ | 0.3 % wt/vol | - | 86 °C |
| DBS-CONHNH$_2$/agarose gel | 0.3 % wt/vol | 1.0 % wt/vol | Above 100 °C |
| Agarose | - | 1.3 % wt/vol | 96 °C |

## S8. Rheology

### *S8.1 Methodology*

Gel samples for rheology were prepared as described in Section S2 using bottomless vials as templates to obtain the intended gel dimensions. The measurements were carried out at 25 °C using a 20 mm parallel plate and a gap of 2 mm. To avoid solvent evaporation and keep the sample hydrated, a solvent trap was used, and the internal atmosphere was kept saturated. Amplitude sweep experiments were performed in the range of 0.05-100 % strain at a 1 Hz frequency to identify the linear viscoelastic region. Frequency sweep experiments were performed between 0.1 and 100 Hz using a shear strain of 0.05 %. The measurements were repeated three times to ensure reproducibility and the average data are shown.

### *S8.2 Rheology data*

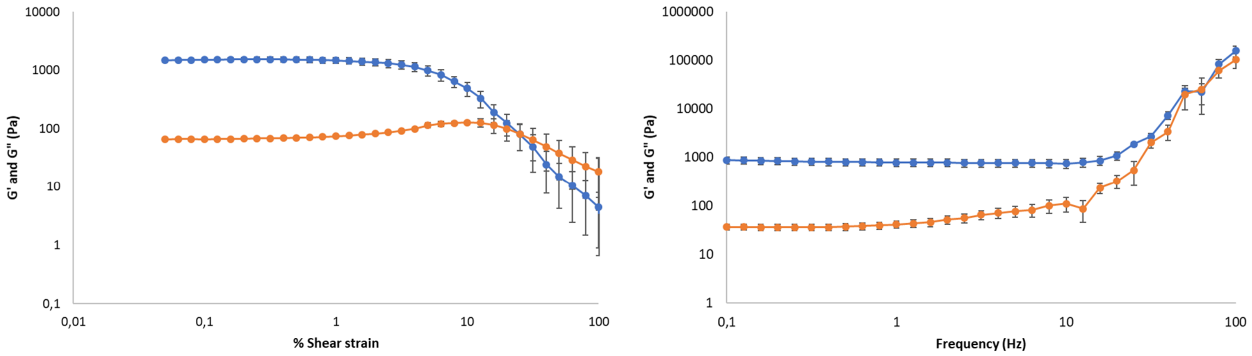


Figure S7. Elastic (G’, blue circles) and viscous (G”, orange circles) moduli of DBS-CONHNH$_2$ hydrogel (0.4 % wt/vol) with increasing shear strain (left) and frequency (right).

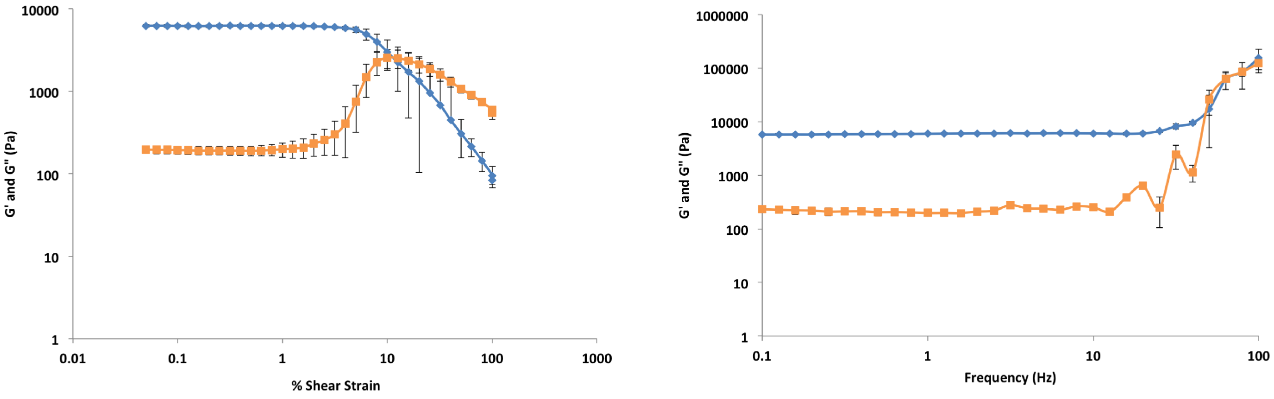


Figure S8. Elastic (G’, blue circles) and viscous (G”, orange circles) moduli of agarose hydrogel (1.0 % wt/vol) with increasing shear strain (left) and frequency (right).

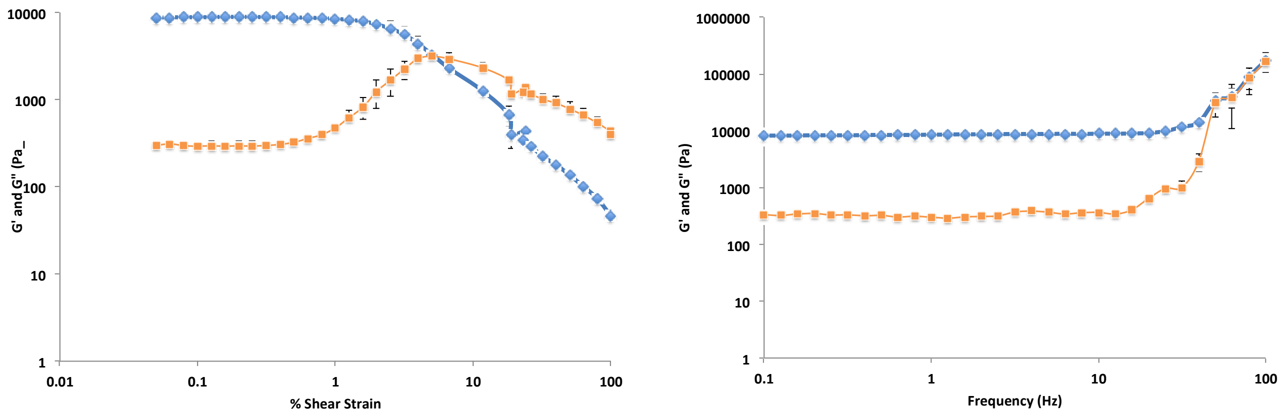


Figure S9. Elastic (G’, blue circles) and viscous (G”, orange circles) moduli of agarose hydrogel (1.3 % wt/vol) with increasing shear strain (left) and frequency (right).

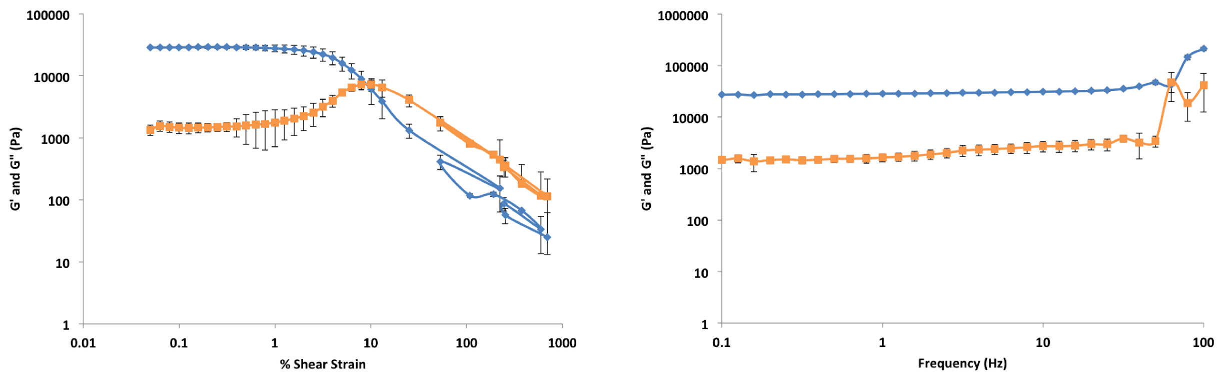


Figure S10. Elastic (G’, blue circles) and viscous (G”, orange circles) moduli of DBS-CONHNH$_2$/agarose hydrogel (0.3 % wt/vol DBS-CONHNH$_2$ and 1.0% wt/vol agarose) with increasing shear strain (left) and frequency (right).

Table S3. Rheological data as determined using oscillatory rheometry with a parallel plate geometry, for DBS-CONHNH$_2$ gels, agarose gels, and hybrid gels formed by the combination of the two. Loadings are given in wt/vol, and the G’/G” crossover points refer to the % shear strain at which G”=G’.

| **Gel** | **Loading of LMWG** | **Loading of Agarose** | **G’ (Pa)** | **G” (Pa)** | **G’/G” Crossover** |
|---|---|---|---|---|---|
| DBS-CONHNH$_2$ | 0.4% | - | 800 | 86 | 25.1% |
| Agarose | - | 1.0% | 5960 | 246 | 12.6% |
| Agarose | - | 1.3% | 8680 | 375 | 5.04% |
| Two-component | 0.3% | 1.0% | 29400 | 2000 | 10.1% |

## S9. Gel beads motion studies

Gel beads for motion studies were prepared as described in Section S2 and immersed in ethanol for 24 hours. The ethanol-loaded gels were isolated and the excess of EtOH was removed with blue paper. The gels were then transferred into a petri dish (8 cm diameter) filled with distilled water. The gels motion in water was recorded with a Xiaomi Redmi Note 7 Mobile Phone and the videos were processed using the *Tracker* Software.

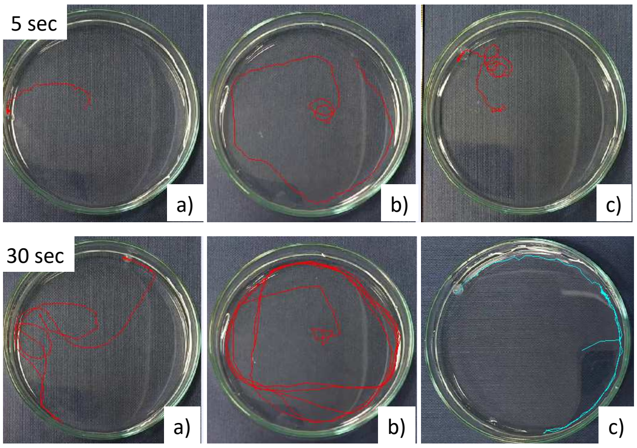


Figure S11. Additional images of trajectories travelled in 5 and 30 sec (respectively upper and lower images) by DBS-CONHNH$_2$/agarose hydrogel beads with: 3.0-3.5 mm diameter (a*), 1.5-2.0 mm diameter (b) and 4.0-4.5 mm diameter (c).

*Gel beads in images a: Only half gel bead was immersed in EtOH for 15 mins.

Table S4. Distance travelled in 5 and 30 sec by DBS-CONHNH$_2$/agarose hydrogel beads with: 3.0-3.5 mm diameter (a and b*), 1.5-2.0 mm diameter (c) and 4.0-4.5 mm diameter (d).

*Gel beads b: Only half gel bead was immersed in EtOH for 15 mins.

| **Hybrid gel beads** | **Distance travelled in 5 sec (cm)** | **Average velocity in 5 sec (cm/s)** | **Distance travelled in 30 sec (cm)** | **Average velocity in 30 sec (cm/s)** |
|---|---|---|---|---|
| **a** | 26.0 | 5.2 | 70.1 | 2.3 |
| **b*** | 12.7 | 2.5 | 31.4 | 1.0 |
| **c** | 25.4 | 5.1 | 102.0 | 3.4 |
| **d** | 15.7 | 3.1 | 27.3 | 0.9 |

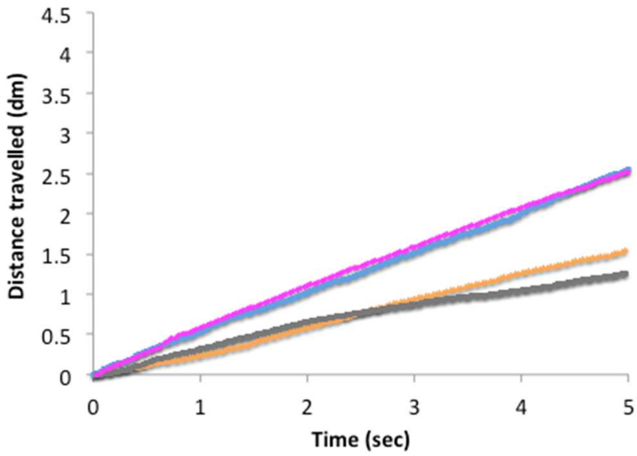


Figure S12. Distance travelled in 5 sec by DBS-CONHNH$_2$/agarose hydrogel beads with: 3.0-3.5 mm diameter (blue and grey lines*), 1.5-2.0 mm diameter (pink line) and 4.0-4.5 mm diameter (orange line). *Grey line: only half gel bead was immersed in EtOH for 15 mins.

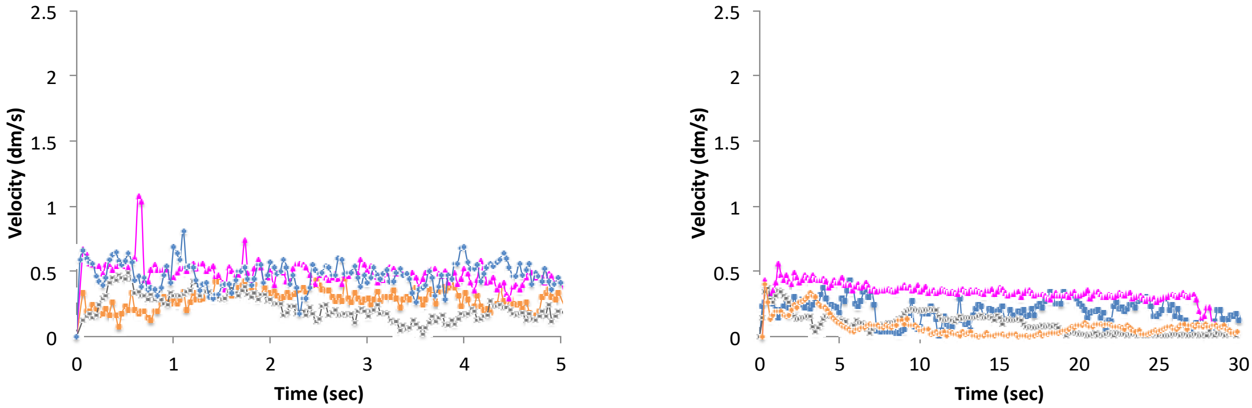


Figure S13. Velocity over time measured for 5 and 30 sec (respectively left and right) by DBS-CONHNH$_2$/agarose hydrogel beads with: 3.0-3.5 mm diameter (blue and grey lines*), 1.5-2.0 mm diameter (pink line) and 4.0-4.5 mm diameter (orange line). *Grey line: only half gel bead was immersed in EtOH for 15 mins.

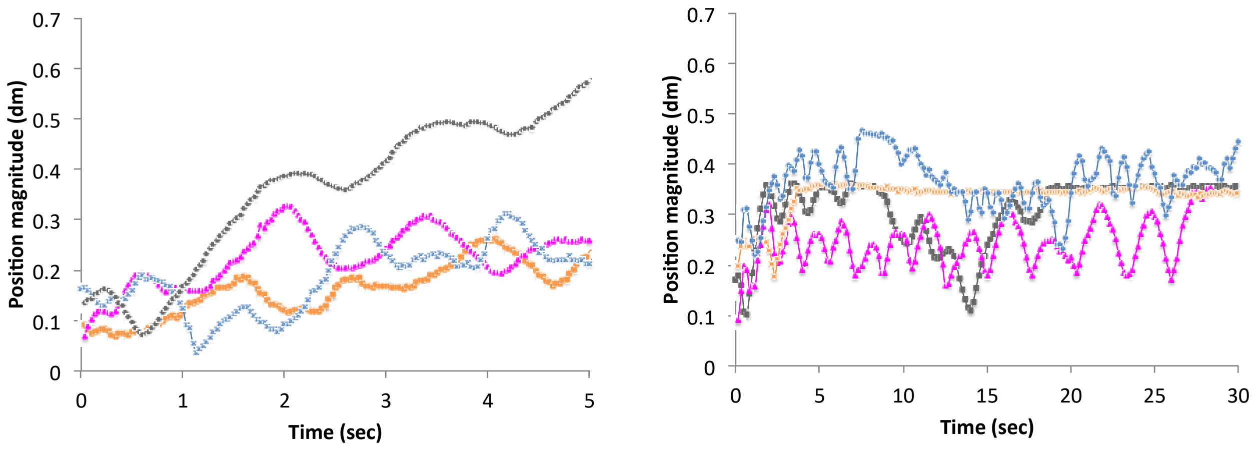


Figure S14. Position magnitude over time measured for 5 and 30 sec (respectively left and right) by DBS-CONHNH$_2$/agarose hydrogel beads with: 3.0-3.5 mm diameter (blue and grey lines*), 1.5-2.0 mm diameter (pink line) and 4.0-4.5 mm diameter (orange line). *Grey line: only half gel bead was immersed in EtOH for 15 mins.

## S10 Ethanol release from gel beads

The amount of ethanol released from each gel was quantified using the ethanol assay (Sigma Aldrich).

### *S10.1 Sample preparation for ethanol assay*

DBS-CONHNH$_2$/agarose and agarose gel beads and shaped gels were prepared as described in section S2. Each gel bead/shaped gel was immersed in EtOH (5 mL) for 24 hours. After 24 hours, the gels were collected with a spatula and left to the air for 5-10 seconds to allow the excess of solvent on the surface to evaporate. Each gel was then immersed in extra pure water (5 mL) for 24 hours. After 30 sec, 60 sec or 24 hours, an aliquot of the supernatant was collected from each sample (25 µL, unless otherwise specified)[*] and diluted with extra pure water to a 0.5 mL final volume. An aliquot of the prepared dilution (50 µL) was subsequently collected and further diluted with extra pure water to a 0.5 mL final volume. A 10 µL aliquot of this second dilution was then transferred to a 96 well plate and diluted with the ethanol assay buffer (40 µL/well). Each sample was prepared in triplicates. Control gel samples, which were not exposed to EtOH, were also analysed. Ethanol standards (0, 2, 4, 6, 8 and 10 nmoles/well) were prepared in duplicates, as described in the assay technical bulletin, and used to obtain an EtOH calibration curve.

---

[*] For bigger gel beads (4-4.5 mm diameter) 12.5 µL aliquots were collected.

*S10.2 Assay reaction*

The Master Reaction mix (50 μL/well; obtained as described in the assay technical bulletin by combining the ethanol probe (2 μL), the ethanol enzyme mix (2 μL) and the ethanol buffer (46 μL)) was added to each sample and EtOH standard. The plate was then covered with foil and incubated at 37 °C for 30 mins. After this time, the absorbance at 570 nm was recorded.

Table S5. EtOH release after 30 sec, 60 sec and 24 hours from DBS-CONHNH$_2$/agarose and agarose gel beads with different diameters. *Only half gel bead was immersed in EtOH for 15 mins.

| Gels | Diameter (mm) | μL of EtOH released (30 sec) | Std error (30 sec) | μL of EtOH released (60 sec) | Std error (60 sec) | μL of EtOH released (24h) | Std error (24h) |
|---|---|---|---|---|---|---|---|
| Hybrid beads | 3.0-3.5 | 4.79 | 0.44 | 8.62 | 1.12 | 19.97 | 0.26 |
| | 3.0-3.5* | 3.01 | 0.48 | 6.15 | 0.06 | 7.32 | 1.100 |
| | 4.0-4.5 | 6.95 | 0.95 | 10.29 | 1.36 | 30.58 | 2.13 |
| | 1.5-2.0 | 3.16 | 0.09 | 6.52 | 0.31 | 11.32 | 0.29 |
| Agarose beads | 3.0-3.5 | 3.37 | 0.19 | 6.35 | 0.04 | 19.39 | 0.19 |
| | 3.0-3.5* | 2.14 | 0.40 | 5.59 | 0.32 | 8.10 | 0.12 |
| | 4.0-4.5 | 5.28 | 0.16 | 7.94 | 0.11 | 31.87 | 1.22 |
| | 1.5-2.0 | 1.74 | 0.59 | 3.95 | 0.35 | 11.97 | 1.03 |

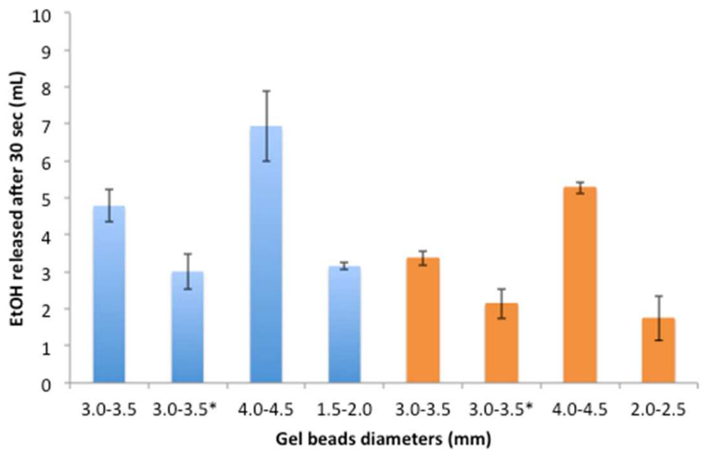


Figure S15. EtOH released in water after 30 sec from DBS-CONHNH$_2$/agarose (blue bars) and agarose gel beads (orange bars) loaded with EtOH.

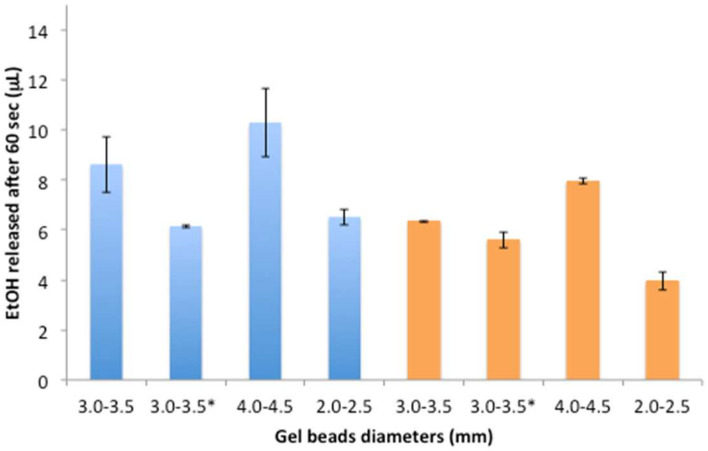


Figure S16. EtOH released in water after 60 sec from DBS-CONHNH$_2$/agarose (blue bars) and agarose gel beads (orange bars) loaded with EtOH.

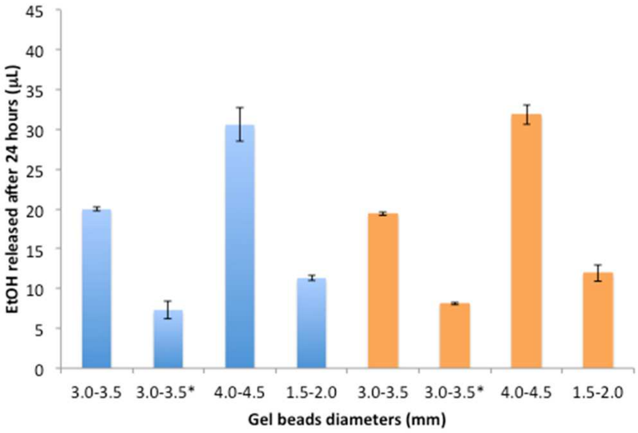


Figure S17. EtOH released in water after 24 hours from DBS-CONHNH$_2$/agarose (blue bars) and agarose gel beads (orange bars) loaded with EtOH.

## S11 Preparation of shaped gels

*S11.1 DBS-CONHNH$_2$/agarose two-component shaped gels.* DBS-CONHNH$_2$ (0.3% wt/vol) and agarose (1.0% wt/vol) were suspended in water (5 mL) and sonicated to help the dispersion of the solid particles. The resulting suspension was heated until complete dissolution of the two gelators. The hot solution was then transferred to a 5 x 5 cm square tray and left undisturbed for 20 mins to allow the formation of the gel. Once a stable gel was obtained, different shapes (*i.e.* stars, crescents and circles) were cut using small icing cutters. The resulting shaped gels were collected and stored in water.

*S11.2 Agarose shaped gels.* Agarose (1.3% wt/vol) was suspended in water (1 mL) and then heated until complete dissolution. The hot solution was transferred to a 5 x 5 cm square tray and left undisturbed for 20 mins to allow the formation of the gel. Once a stable gel was obtained, different shapes (*i.e.* stars, crescents and circles) were cut using small icing cutters. The resulting shaped gels were collected and stored in water.

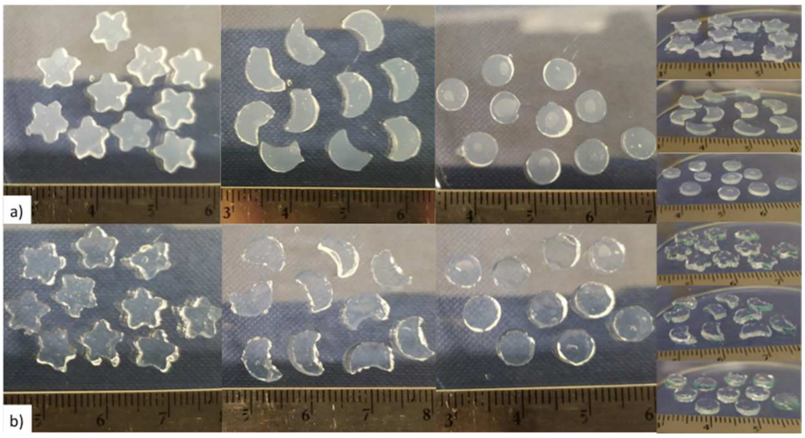

Figure S18. a) DBS-CONHNH$_2$/agarose shaped gels (prepared with 0.3% wt/vol DBS-CONHNH$_2$ and 1.0% wt/vol agarose); b) agarose shaped gels (1.3% wt/vol).

Table S6. Average dimensions and weights of DBS-CONHNH$_2$/agarose shaped gels (prepared with 0.3% wt/vol DBS-CONHNH$_2$ and 1.0% wt/vol agarose) and agarose shaped gels (1.3% wt/vol).

| Gel | Average Length (mm) | Average Thickness (mm) | Average Weight (mg) |
|---|---|---|---|
| Hybrid stars | 5.58 +/- 0.20 | 0.86 +/- 0.10 | 33.34 +/- 0.91 |
| Hybrid crescents | 6.55 +/- 0.40 | 0.90 +/- 0.14 | 33.67 +/- 0.91 |
| Hybrid circles | 5.39 +/- 0.23 | 0.88 +/- 0.16 | 27.19 +/- 0.99 |
| Agarose stars | 5.40 +/- 0.20 | 1.01 +/- 0.14 | 36.34 +/- 0.44 |
| Agarose crescents | 6.38 +/- 0.35 | 1.12 +/- 0.04 | 37.07 +/- 0.97 |
| Agarose circles | 5.22 +/- 0.30 | 1.05 +/- 0.14 | 30.46 +/- 0.93 |

*S11.3 Preparation of shaped gels for motion studies.* DBS-CONHNH$_2$/agarose and agarose shaped gels for gel motion studies were prepared as described in the sections above and immersed in EtOH for 24 hours. The ethanol-loaded gels were isolated and the excess of EtOH was removed with blue paper.

## S12. Shaped gel motion studies

Shaped gels for motion studies were prepared as described in Section S11. The ethanol-loaded gels were transferred into a petri dish (8 cm diameter) filled with distilled water. The gels motion in water was recorded with a Xiaomi Redmi Note 7 Mobile Phone and the videos were processed using the *Tracker* Software.

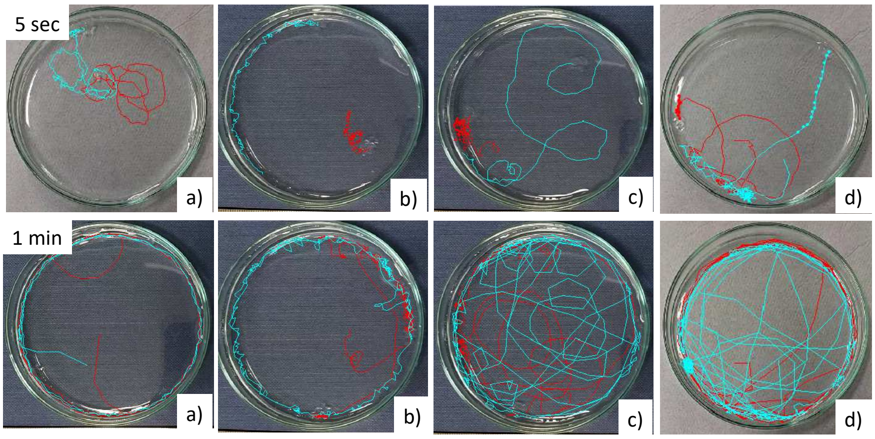


Figure S19. Trajectories travelled in 5 and 60 sec (respectively upper and lower images) by: agarose hydrogel beads (3.0-3.5 mm diameter; a), agarose star-shaped gels (b), agarose crescent-shaped gels (c) and agarose circle-shaped gels (d).

Table S7. Distance travelled and speed in 5 and 60 sec by DBS-$CONHNH_2$/agarose two-component gels and agarose gels.

| **Gels** | **Distance travelled in 5 sec (cm - left) and speed (cm/s - right)** | | | | **Distance travelled in 60 sec (cm - left) and speed (cm/s - right)** | | | |
|---|---|---|---|---|---|---|---|---|
| | **DBS-$CONHNH_2$/Agarose** | | **Agarose** | | **DBS-$CONHNH_2$/Agarose** | | **Agarose** | |
| **Beads** | 26.0 | 5.2 | 41.9 | 8.3 | 127 | 2.1 | 61.0 | 1.0 |
| **Stars** | 21.7 | 4.3 | 14.9 | 3.0 | 104 | 1.7 | 60.9 | 1.0 |
| **Crescents** | 28.0 | 5.6 | 30.4 | 6.1 | 215 | 3.6 | 165 | 2.7 |
| **Circles** | 29.0 | 5.8 | 32.7 | 6.5 | 324 | 5.4 | 206 | 3.4 |

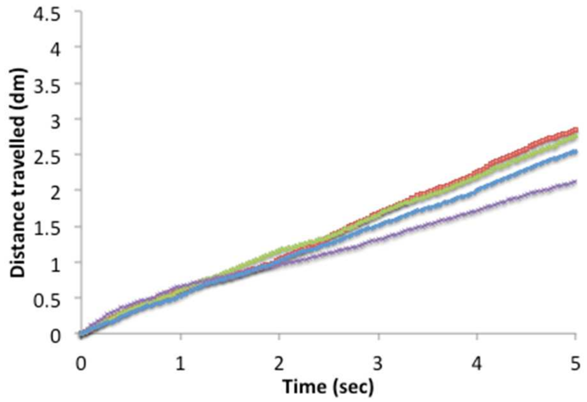


Figure S20. Distance travelled in 5 sec by DBS-$CONHNH_2$/agarose gel beads (3.0-3.5 mm diameter, blue line) and DBS-$CONHNH_2$/agarose shaped gels (star, purple; crescent, green; circle, red).

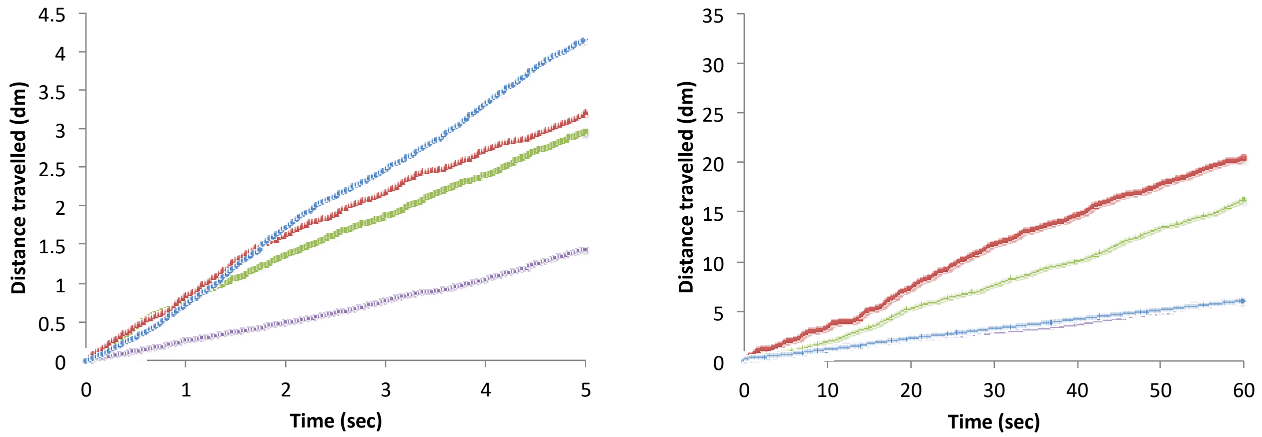


Figure S21. Distance travelled in 5 and 60 sec (left and right) by agarose gel beads (3.0-3.5 mm diameter, blue line) and agarose shaped gels (star, purple; crescent, green; circle, red).

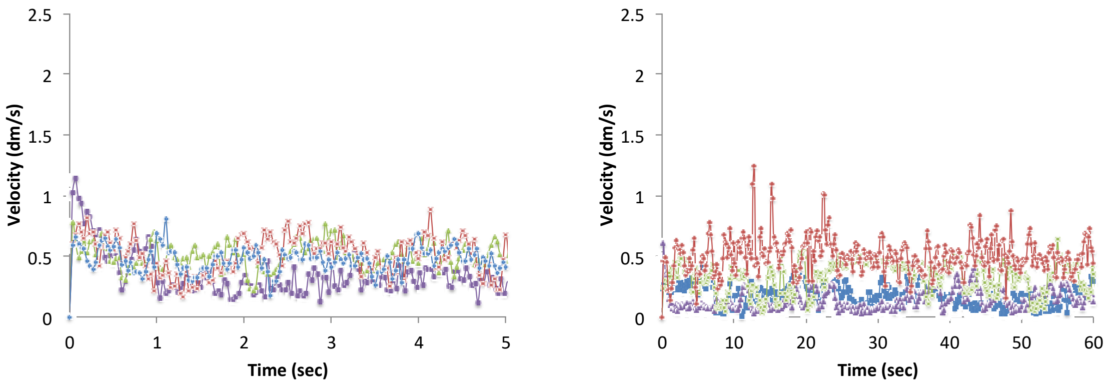


Figure S22. Velocity over time measured for 5 and 60 sec (left and right) by DBS-$CONHNH_2$/agarose gel beads (3.0-3.5 mm diameter, blue) and DBS-$CONHNH_2$/agarose shaped gels (star, purple; crescent, green; circle, red).

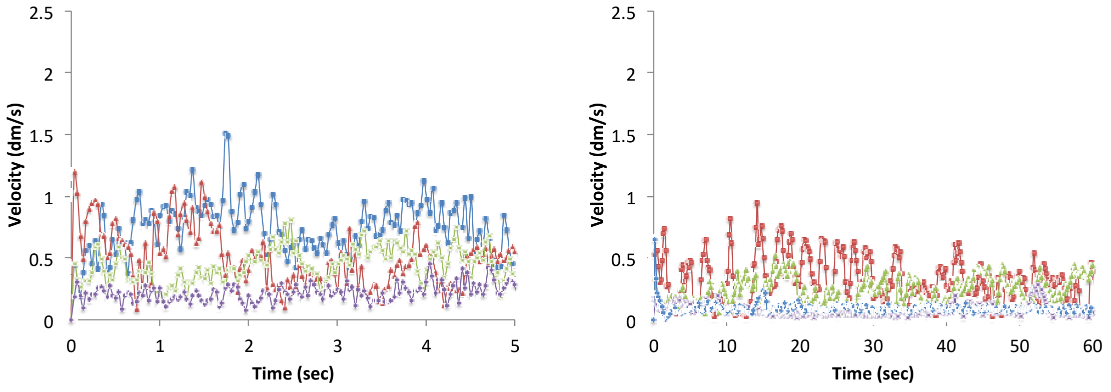


Figure S23. Velocity over time measured for 5 and 60 sec (left and right) by agarose gel beads (3.0-3.5 mm diameter, blue) and agarose shaped gels (star, purple; crescent, green; circle, red).

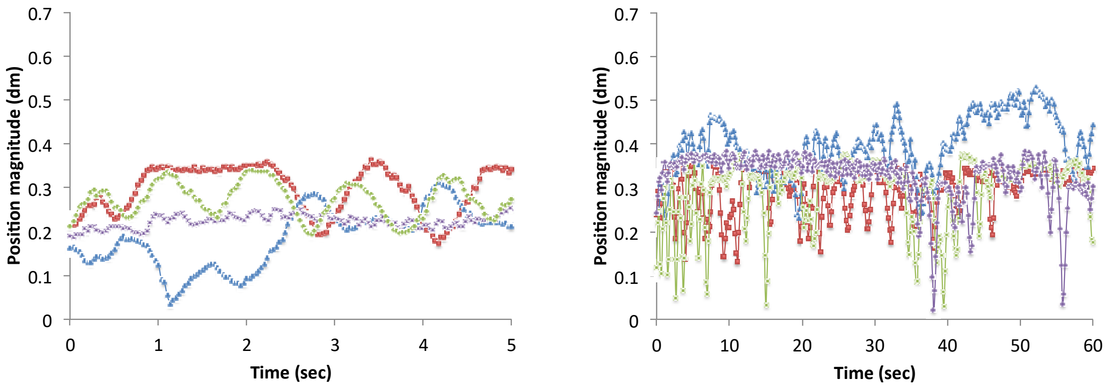


Figure S24. Position magnitude over time measured for 5 and 60 sec (left and right) by DBS-CONHNH$_2$/agarose gel beads (3.0-3.5 mm diameter, blue) and DBS-CONHNH$_2$/agarose shaped gels (star, purple; crescent, green; circle, red).

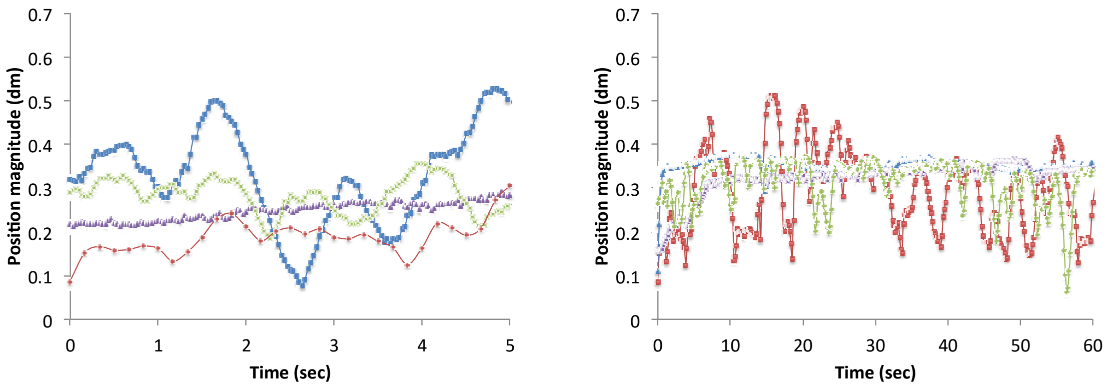


Figure S25. Position magnitude over time measured for 5 and 60 sec (left and right) by agarose gel beads (3.0-3.5 mm diameter, blue) and agarose shaped gels (star, purple; crescent, green; circle, red).

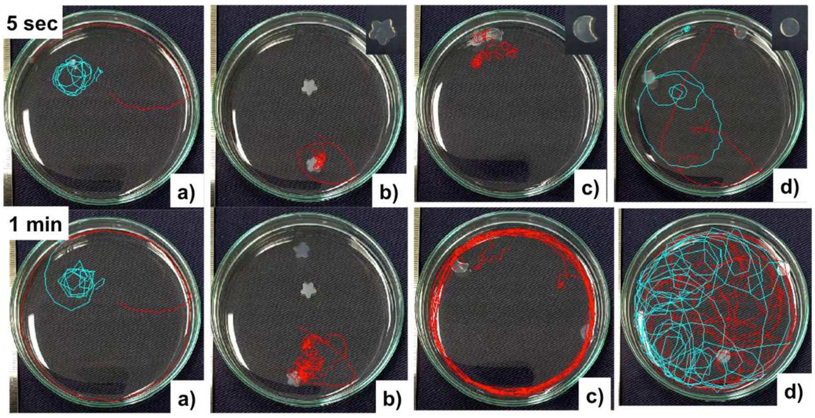


Figure S26. Trajectories travelled in a NaOH 0.1 M solution (pH 11.8-12) in 5 and 60 sec (respectively upper and lower images) by DBS-CONHNH$_2$/agarose: (a) gel beads (3.0-3.5 mm diameter; (b) star-shaped gels; (c) crescent-shaped gels; (d) circle-shaped gels.

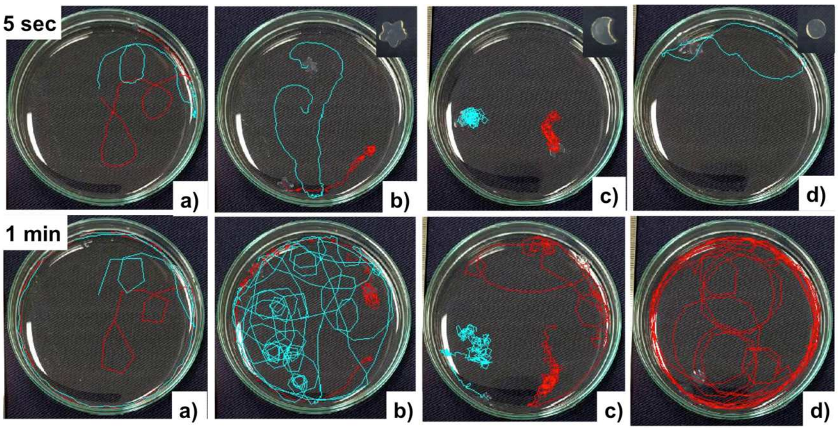


Figure S27. Trajectories travelled in a NaOH 0.1 M solution (pH 11.8-12) in 5 and 60 sec (respectively upper and lower images) by agarose: (a) gel beads (3.0-3.5 mm diameter; (b) star-shaped gels; (c) crescent-shaped gels; (d) circle-shaped gels.

Table S8. Distance travelled in a NaOH 0.1 M solution (pH 11.8-12) and speed in 5 and 60 sec by DBS-CONHNH$_2$/agarose two-component gels and agarose gels.

| **Gels** | **Distance travelled in 5 sec (cm - left) and speed (cm/s - right)** | | | | **Distance travelled in 60 sec (cm - left) and speed (cm/s - right)** | | | |
|---|---|---|---|---|---|---|---|---|
| | **DBS-CONHNH$_2$/Agarose** | | **Agarose** | | **DBS-CONHNH$_2$/Agarose** | | **Agarose** | |
| **Beads** | 17.5 | 3.50 | 16.5 | 3.30 | 34.0* | 0.57 | 40.0* | 0.67 |
| **Stars** | 16.3 | 3.26 | 15.9 | 3.18 | 142 | 2.36 | 108 | 1.80 |
| **Crescents** | 16.8 | 3.36 | 26.8 | 5.36 | 191 | 3.18 | 83.1 | 1.38 |
| **Circles** | 23.6 | 4.72 | 15.2 | 3.04 | 260 | 4.33 | 281 | 4.68 |

*The DBS-CONHNH$_2$/agarose gel beads and agarose gel beads stopped after ca. 20sec.

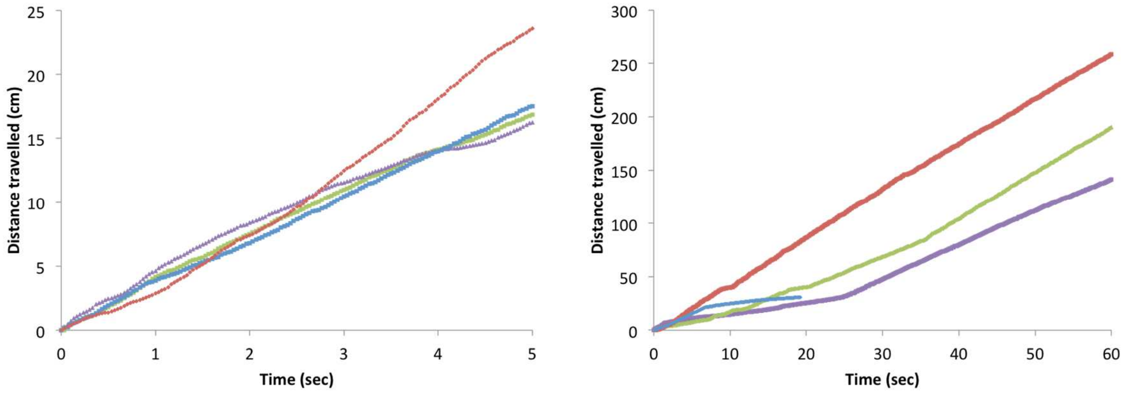


Figure S28. Distance travelled in a NaOH 0.1 M solution (pH 11.8-12) in 5 and 60 sec by DBS-$CONHNH_2$/agarose gel beads (3.0-3.5 mm diameter, blue line) and DBS-$CONHNH_2$/agarose shaped gels (star, purple; crescent, green; circle, red).

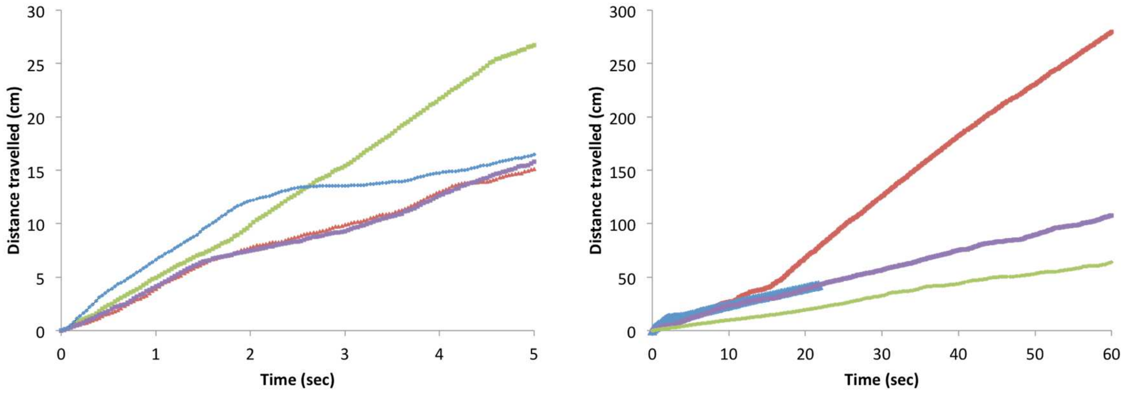


Figure S29. Distance travelled in a NaOH 0.1 M solution (pH 11.8-12) in 5 and 60 sec (left and right) by agarose gel beads (3.0-3.5 mm diameter, blue line) and agarose shaped gels (star, purple; crescent, green; circle, red).

## S13 Ethanol release from shaped gels

The amount of ethanol released from each gel was quantified using the ethanol assay (Sigma Aldrich), as described in Section S10.

Table S9. EtOH release after 30 sec, 60 sec and 24 hours from DBS-CONHNH$_2$/agarose and agarose gel beads and shaped gels.

| Gels | Shape | µL of EtOH released (30 sec) | Std error (30 sec) | µL of EtOH released (60 sec) | Std error (60 sec) | µL of EtOH released (24h) | Std error (24h) |
|---|---|---|---|---|---|---|---|
| Hybrid | Beads | 4.79 | 0.44 | 8.62 | 1.12 | 19.97 | 0.26 |
| | Stars | 4.83 | 0.03 | 9.0 | 0.42 | 32.18 | 3.27 |
| | Crescents | 4.52 | 0.08 | 7.86 | 0.52 | 32.14 | 1.89 |
| | Circles | 4.56 | 0.54 | 8.67 | 0.53 | 31.33 | 2.38 |
| Agarose | Beads | 3.37 | 0.19 | 6.35 | 0.04 | 19.39 | 0.19 |
| | Stars | 4.27 | 0.30 | 7.36 | 0.85 | 34.08 | 3.56 |
| | Crescents | 3.65 | 0.56 | 6.29 | 0.50 | 37.20 | 0.92 |
| | Circles | 4.32 | 0.12 | 6.64 | 0.21 | 28.08 | 1.01 |

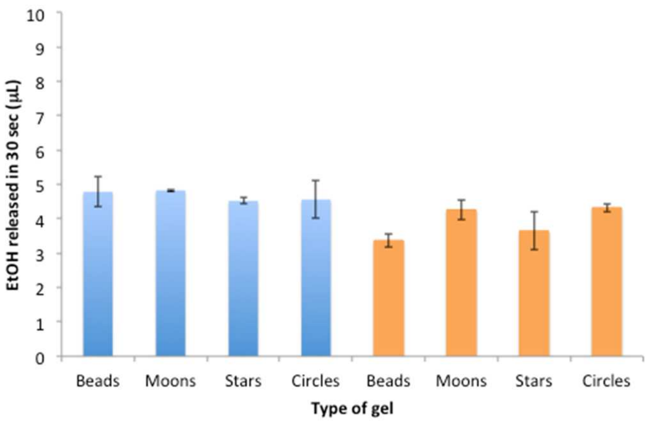


Figure S30. EtOH released in water after 30 sec from DBS-CONHNH$_2$/agarose and agarose shaped gels loaded with EtOH (moons = crescents).

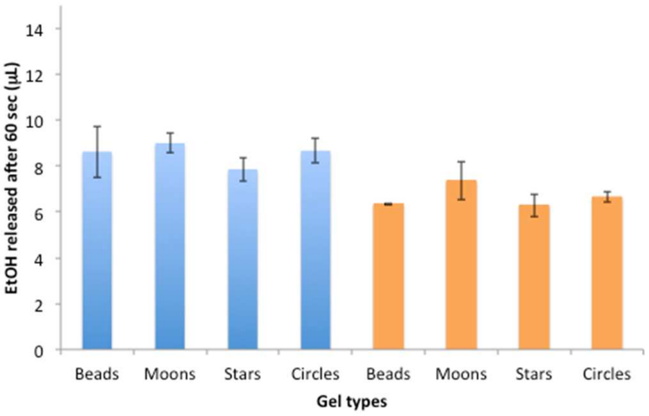


Figure S31. EtOH released in water after 60 sec from DBS-CONHNH$_2$/agarose and agarose shaped gels loaded with EtOH (moons = crescents).

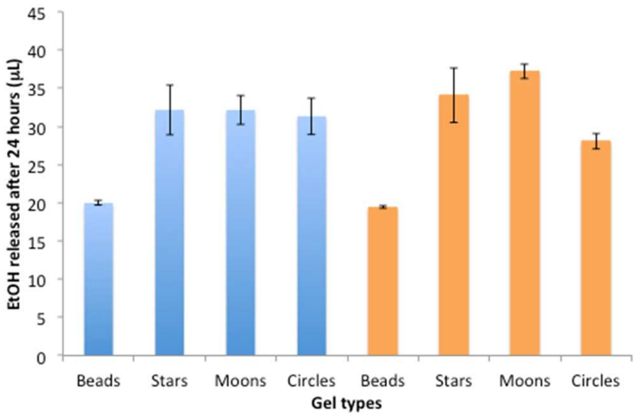


Figure S32. EtOH released in water after 24 hours from DBS-CONHNH$_2$/agarose and agarose gel beads and shaped gels loaded with EtOH (moons = crescents).

## S14 Dye uptake studies

*S14.1 Maximum dye uptake under different pH conditions*

DBS-CONHNH$_2$/agarose and agarose gel beads were prepared as described in Section S2. Fifteen beads were immersed in 5 mL of a concentrated methylene blue solution (3.67 mM) at pH 11.8-12, 7 or 2.8 and left undisturbed for 24 hours. After 24 hours, a 6.25 µL aliquot of the dye was collected and diluted with water (2 mL total volume) into a cuvette. The UV-vis absorbance at 662 nm was recorded. To ensure reproducibility, the data were collected in triplicate and the average is shown.

Table S10. Methylene blue maximum uptake under different pH conditions by DBS-CONHNH$_2$/agarose gel beads (prepared with 0.3 % wt/vol of LMWG and 1.0% wt/vol of PG) and agarose gel beads (1.3 % wt/vol).

| Gels | Methylene blue uptake (mg/g of gelator) | | |
|---|---|---|---|
| | **pH 11.8-12** | **pH 7** | **pH 2.8** |
| **DBS-CONHNH$_2$/Agarose gel beads** | 174.09 | 12.02 | 22.34 |
| **Agarose gel beads** | 63.69 | 5.29 | 24.35 |

*S14.2 Methylene blue uptake rate under different pH conditions*

DBS-CONHNH$_2$/agarose and agarose gel beads (15 beads/sample) were immersed in 5 mL of a methylene blue solution (0.018 mM) at pH 11.8-12, 7 or 2.8. At regular time intervals, 2 mL of the dye solution were transferred into a cuvette and the UV-vis absorbance at 662 nm was recorded. To ensure reproducibility, the data were collected in triplicate and the average is shown.

Table S11. Methylene blue uptake (%) over time by DBS-CONHNH$_2$/agarose and agarose gel beads under different pH conditions.

| Time (hours) | Methylene blue uptake (%) | | | | | | | | |
|---|---|---|---|---|---|---|---|---|---|
| | DBS-CONHNH$_2$ | | | DBS-CONHNH$_2$/Agarose gel beads | | | Agarose gel beads | | |
| | pH 11.8-12 | pH 7 | pH 2.8 | pH 11.8-12 | pH 7 | pH 2.8 | pH 11.8-12 | pH 7 | pH 2.8 |
| 0 | 0 | 0 | 0 | 0 | 0 | 0 | 0 | 0 | 0 |
| 1 | 19.62 | 7.61 | 6.37 | 14.91 | 12.04 | 4.63 | 11.92 | 8.38 | 17.82 |
| 2 | 33.08 | 15.25 | 7.57 | 20.49 | 14.80 | 9.10 | 13.31 | 10.91 | 18.91 |
| 3 | - | 17.26 | 8.92 | - | 15.31 | 9.32 | - | 11.85 | 19.02 |
| 4 | 36.11 | 18.651 | 11.57 | 25.67 | 16.06 | 9.64 | 15.17 | 12.66 | 19.50 |
| 5 | 36.35 | 19.33 | 12.70 | 28.19 | 16.58 | 9.97 | 15.90 | 13.50 | 20.00 |
| 6 | 35.99 | 19.75 | 13.21 | 31.18 | 17.03 | 10.01 | 17.43 | 14.12 | 19.64 |
| 7 | 38.17 | 19.95 | 13.75 | 33.77 | 17.74 | 10.37 | 18.03 | 14.73 | 19.93 |
| 24 | 38.54 | 24.19 | 14.90 | 37.96 | 20.04 | 10.92 | 18.76 | 18.59 | 20.73 |

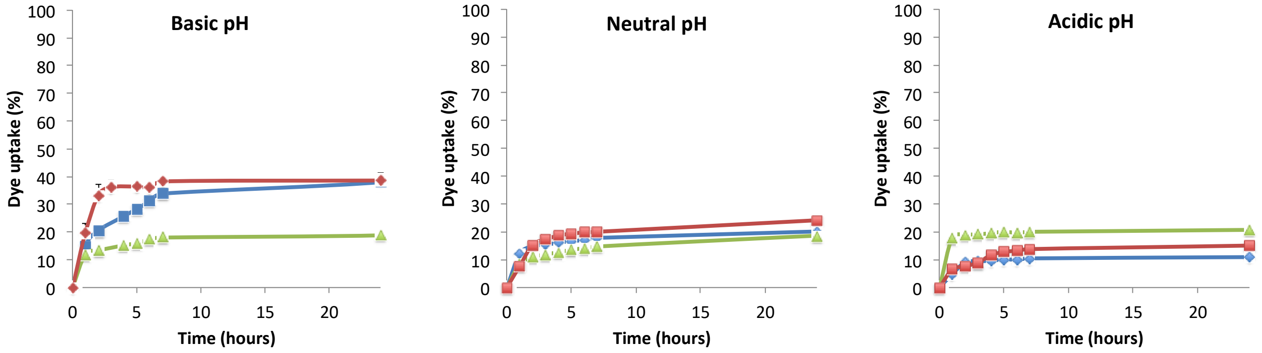


Figure S33. Uptake of methylene blue (%) over time at pH 11.8-12 (left), 7.0-7.2 (centre) and 2.8 (right) by: DBS-CONHNH$_2$ gels (0.3 % wt/vol – red line), DBS-CONHNH$_2$/agarose gel beads (prepared with 0.3 % wt/vol of LMWG and 1.0% wt/vol of PG – blue line) and agarose gel beads (1.3 % wt/vol – green line).

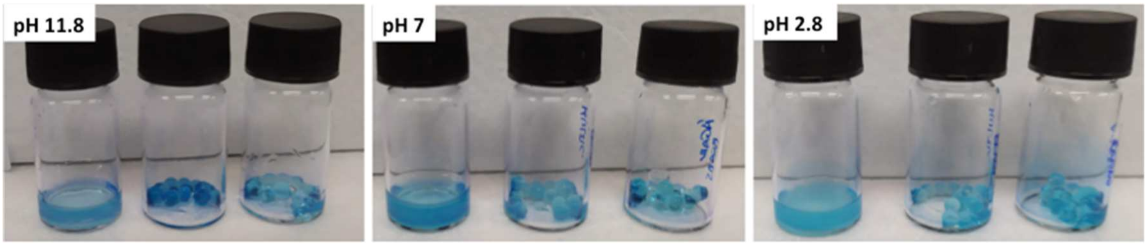


Figure S34. DBS-CONHNH$_2$ gels (0.3 % wt/vol – left), DBS-CONHNH$_2$/agarose gel beads (prepared with 0.3 % wt/vol of LMWG and 1.0% wt/vol of PG – centre) and agarose gel beads (1.3 % wt/vol – right) after methylene blue uptake study (24 hours).

*S14.3 Methylene blue uptake at basic pH - recyclability studies*

DBS-CONHNH$_2$/agarose and agarose gel beads (15 beads/sample) were immersed in 5 mL of a methylene blue solution (0.020 mM) at pH 11.8-12. At regular time intervals, 2 mL of the dye solution were transferred into a cuvette and the UV-vis absorbance at 662 nm was recorded. After 24 hours, the gel beads were collected and washed multiple times with a NaOH solution (0.5 M) until the captured dye was completely removed. The clean beads were then re-used in a subsequent dye uptake cycle. This procedure was repeated four times. To ensure reproducibility, the data were collected in triplicate and the average is shown.

Table S12. Methylene blue uptake (%) over time at pH 11.8-12 by DBS-CONHNH$_2$/agarose and agarose gel beads in subsequent cycles performed using the same gel beads.

| Time (hours) | Methylene blue uptake (%) – Recyclability study | | | | | | | |
|---|---|---|---|---|---|---|---|---|
| | DBS-CONHNH$_2$/Agarose gel beads | | | | Agarose gel beads | | | |
| | 1$^{st}$ cycle | 2$^{nd}$ cycle | 3$^{rd}$ cycle | 4$^{th}$ cycle | 1$^{st}$ cycle | 2$^{nd}$ cycle | 3$^{rd}$ cycle | 4$^{th}$ cycle |
| 0 | 0 | 0 | 0 | 0 | 0 | 0 | 0 | 0 |
| 1 | 14.91 | 9.32 | 9.88 | 27.02 | 11.92 | 5.26 | 2.00 | 23.75 |
| 2 | 20.49 | 22.93 | 26.16 | 36.84 | 13.31 | 10.62 | 6.72 | 27.62 |
| 3 | - | 28.86 | 35.72 | 39.63 | - | 11.64 | 12.35 | - |
| 4 | 25.67 | 33.32 | 42.31 | - | 15.17 | 11.10 | 18.07 | 31.63 |
| 5 | 28.19 | 36.53 | 48.40 | 45.93 | 15.90 | 12.62 | 22.96 | 28.35 |
| 6 | 31.18 | 38.10 | 53.25 | 50.42 | 17.43 | 13.25 | 26.03 | 26.17 |
| 7 | 33.77 | 39.70 | 53.66 | 50.05 | 18.03 | 14.18 | 24.88 | 26.65 |
| 24 | 37.96 | 46.75 | 52.88 | 60.48 | 18.76 | 18.95 | 24.01 | 27.14 |

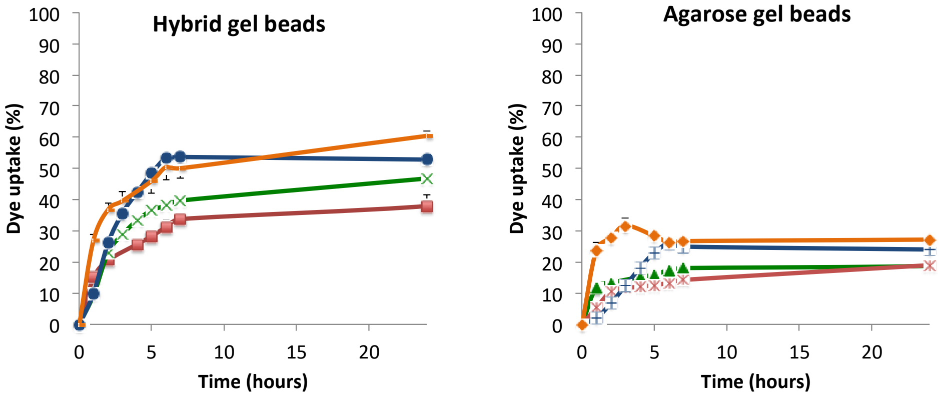


Figure S35. Recyclability studies showing the methylene blue (pH 11.8-12) uptake (%) over time by DBS-CONHNH$_2$/agarose gel beads (prepared with 0.3 % wt/vol of LMWG and 1.0% wt/vol of PG) and agarose gel beads (1.3 % wt/vol) in four different cycles using the same beads (1$^{st}$ cycle – red line, 2$^{nd}$ cycle green line, 3$^{rd}$ cycle blue line and 4$^{th}$ cycle orange line).

*S14.4 Methylene blue increased uptake at basic pH by substitution or addition of new gel beads*

To increase dye uptake, two different experimental settings were tested by (a) replacing the gel beads in each sample with new beads or (b) by adding extra gel beads after 3, 5 and 7 hours. The two experiments were performed as follows:

a) DBS-CONHNH$_2$/agarose and agarose gel beads (15 beads/sample) were immersed in 5 mL of a methylene blue solution (0.018 mM) at pH 11.8-12. Every hour for the first 7 hours, the gel beads were collected and replaced with new beads (15 beads/sample).

b) DBS-CONHNH$_2$/agarose and agarose gel beads (15 beads/sample) were immersed in 5 mL of a methylene blue solution (0.018 mM) at pH 11.8-12. After 3, 5 and 7 hours, 15 new extra beads were added to each sample, which at the end of the study comprised 60 beads.

In both experimental settings, at regular time intervals, 2 mL of the dye solution were transferred into a cuvette and the UV-vis absorbance at 662 nm was recorded. To ensure reproducibility, the data were collected in triplicate and the average is shown.

Table S13. Methylene blue uptake (%) over time at pH 11.8-12 by DBS-CONHNH$_2$/agarose and agarose gel beads using different experimental settings: 1) Experimental conditions described in Section S11.2 (*i.e.* the samples were left undisturbed for the entire duration of the study); 2) Every hour the gel beads were replaced with new gel beads; 3) After 3, 5 and 7 hours, 15 new extra gel beads were added to each sample.

| Time (hours) | Methylene blue uptake (%) | | | | | |
|---|---|---|---|---|---|---|
| | DBS-CONHNH$_2$/Agarose gel beads | | | Agarose gel beads | | |
| | Experimental setting 1 | Experimental setting 2 | Experimental setting 3 | Experimental setting 1 | Experimental setting 2 | Experimental setting 3 |
| 0 | 0 | 0 | 0 | 0 | 0 | 0 |
| 1 | 14.91 | 9.79 | 9.53 | 11.92 | 6.40 | 5.78 |
| 2 | 20.49 | 21.00 | 18.29 | 13.31 | 14.28 | 11.46 |
| 3 | - | 33.10 | 22.41 | - | 23.25 | 12.71 |
| 4 | 25.67 | 43.58 | 29.14 | 15.17 | 28.46 | 17.15 |
| 5 | 28.19 | 50.99 | 33.36 | 15.90 | 35.97 | 18.03 |
| 6 | 31.18 | 56.98 | 37.43 | 17.43 | 43.37 | 22.62 |
| 7 | 33.77 | 60.27 | 40.59 | 18.03 | 48.64 | 23.56 |
| 24 | 37.96 | 68.24 | 46.71 | 18.76 | 51.04 | 26.37 |

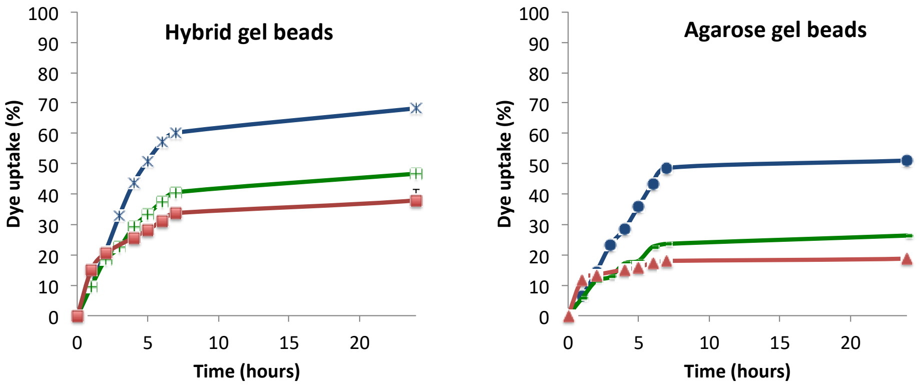


Figure S36. Methylene blue uptake (%) over time at pH 11.8-12 by DBS-CONHNH$_2$/agarose and agarose gel beads using different experimental settings: 1) Experimental conditions described in Section S10.2 (*i.e.* the samples were left undisturbed for the entire duration of the study; red line); 2) Every hour the gel beads were replaced with new gel beads (blue line); 3) After 3, 5 and 7 hours, 15 new extra gel beads were added to each sample (green line).

### *S14.5 Methylene blue uptake at basic pH by static gel beads and shaped gels*

The DBS-CONHNH$_2$/agarose and agarose gel beads and shaped gels used in this experiment were prepared as described in Section S2. Each sample (15 beads or shaped gels/sample) was immersed in 5 mL of an aqueous methylene blue solution (0.016 mM) at pH 11.8-12. At regular time intervals, 2 mL of the dye solution were transferred into a cuvette and the UV-vis absorbance at 662 nm was recorded. To ensure reproducibility, the data were collected in triplicate and the average is shown.

Table S14. Methylene blue uptake (%) over time at pH 11.8-12 by DBS-CONHNH$_2$/agarose and agarose gel beads and shaped gels.

| Time (hours) | Methylene blue uptake (%) | | | | | | | |
|---|---|---|---|---|---|---|---|---|
| | DBS-CONHNH$_2$/Agarose gels | | | | Agarose gels | | | |
| | Beads | Circles | Crescents | Stars | Beads | Circles | Crescents | Stars |
| 0 | 0 | 0 | 0 | 0 | 0 | 0 | 0 | 0 |
| 0.25 | 12.02 | 23.91 | 11.94 | 3.96 | 10.05 | 16.23 | 3.05 | 2.03 |
| 0.5 | 14.25 | 32.54 | 14.04 | 5.71 | 11.47 | 20.18 | 3.70 | 3.87 |
| 1 | 14.91 | 43.34 | 14.80 | 13.11 | 11.92 | 38.74 | 7.61 | 7.83 |
| 2 | 20.49 | 47.90 | 21.50 | 18.61 | 13.31 | 43.13 | 9.35 | 9.62 |
| 3 | - | - | 25.42 | 22.86 | - | - | 10.22 | 10.55 |
| 4 | 25.67 | 39.41 | 29.66 | 25.96 | 15.17 | 33.88 | 11.26 | 11.20 |
| 5 | 28.19 | 39.36 | 33.20 | 32.01 | 15.90 | 31.76 | 12.67 | 13.76 |
| 6 | 31.18 | 39.88 | 37.99 | 37.67 | 17.43 | 30.16 | 13.98 | 10.28 |
| 7 | 33.77 | 40.08 | 41.97 | 38.59 | 18.03 | 29.43 | 14.85 | 10.55 |
| 24 | 37.96 | 50.89 | - | - | 18.76 | 36.67 | - | - |
| 28 | 42.67 | 55.1 | 48.61 | 42.13 | 20.88 | 39.05 | 21.44 | 13.54 |

*S14.6 Methylene blue uptake at basic pH by DBS-CONHNH$_2$/agarose and agarose gel beads and shaped gels in motion (i.e. gels loaded with ethanol)*

The DBS-CONHNH$_2$/agarose and agarose shaped gels used in this experiment were prepared as described in Section S2 and immersed in EtOH for 24 hours. Each sample (containing 15 gel beads or shaped gels) was transferred to a 5 x 5 cm square tray containing 5 mL of an aqueous methylene blue solution (0.016 mM) at pH 11.8-12. To avoid solvent evaporation, the trays were sealed with parafilm and covered with a petri dish. When the gels stopped moving (after 1h), they were transferred into vials. At regular time intervals, 2 mL of the dye solution was transferred into a cuvette and the UV-vis absorbance at 662 nm was recorded. To ensure reproducibility, the data were collected in triplicate and the average is shown.

Table S15. Methylene blue uptake (%) over time at pH 11.8-12 by DBS-CONHNH$_2$/agarose and agarose gel beads and shaped gels loaded with EtOH.

| Time (hours) | Methylene blue uptake (%) | | | | | | | |
|---|---|---|---|---|---|---|---|---|
| | DBS-CONHNH$_2$/Agarose gels loaded with EtOH | | | | Agarose gels loaded with EtOH | | | |
| | Beads | Circles | Crescents | Stars | Beads | Circles | Crescents | Stars |
| 0 | 0 | 0 | 0 | 0 | 0 | 0 | 0 | 0 |
| 0.25 | 7.47 | 16.87 | 5.76 | 0.99 | 4.81 | 11.98 | 2.24 | 1.17 |
| 0.5 | 9.53 | 20.78 | 8.85 | 3.10 | 6.28 | 12.84 | 3.23 | 1.68 |
| 1 | 13.02 | 26.12 | 16.40 | 7.20 | 9.54 | 23.95 | 12.93 | 3.52 |
| 2 | 15.45 | 29.95 | 21.39 | 10.59 | 8.80 | 26.18 | 16.34 | 2.18 |
| 3 | - | - | - | 14.23 | - | - | - | 2.66 |
| 4 | 17.99 | 34.09 | 25.80 | 17.00 | 10.48 | 30.16 | 15.77 | 3.33 |
| 5 | 19.43 | 34.86 | 28.14 | 22.07 | 11.35 | 30.93 | 18.23 | 4.24 |
| 6 | 22.54 | 36.67 | 31.17 | 25.08 | 12.40 | 33.31 | 19.81 | 5.43 |
| 7 | 23.78 | 37.39 | 33.82 | 26.08 | 13.46 | 33.93 | 20.69 | 6.29 |
| 24 | 26.20 | 45.56 | 38.17 | - | 13.52 | 39.82 | 20.12 | - |
| 28 | 30.18 | 48.20 | 42.21 | 33.44 | 15.45 | 42.51 | 23.59 | 7.54 |

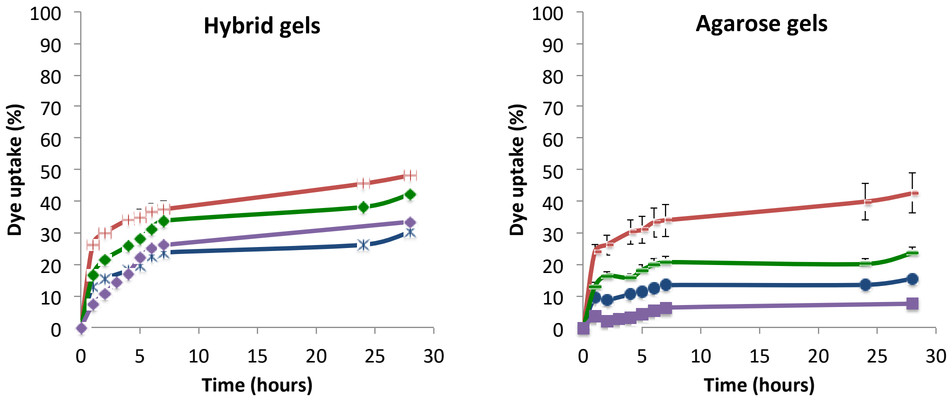


Figure S37. Methylene blue uptake (%) over time at pH 11.8-12 by DBS-CONHNH$_2$/agarose (prepared with 0.3 % wt/vol of LMWG and 1.0% wt/vol of PG) and agarose (1.3 % wt/vol) gel beads (blue line) and shaped gels (circles, red line; crescents, green line; stars, purple line) loaded with EtOH.

## S15 References


1. B. O. Okesola and D. K. Smith, *Chemical Communications*, 2013, **49**, 11164-11166.
2. D. J. Cornwell, B. O. Okesola and D. K. Smith, *Soft Matter*, 2013, **9**, 8730-8736.
